# Carboxyhemoglobin and depletion of blood oxygen in sleeping elephant seals

**DOI:** 10.1007/s00360-026-01680-1

**Published:** 2026-06-06

**Authors:** P. J. Ponganis, B. I. McDonald, C. L. Williams, J. U. Meir, C. V. Brown, A. Patrician, J. C. Tremblay, A. G. Hindle, L. J. Pallin, J. M. Kendall-Bar, J. C. McKnight, D. P. Costa, T. M. Williams, P. N. Ainslie

**Affiliations:** 1 Center for Marine Biotechnology & Biomedicine, Scripps Institution of Oceanography, University of California San Diego, La Jolla, CA 92093-0204 USA; 2 Moss Landing Marine Laboratories, California State University, 8272 Moss Landing Road, Moss Landing, CA 95039 USA; 3 National Marine Mammal Foundation, 2240 Shelter Island Drive, San Diego, CA 92106 USA; 4 NASA, Johnson Space Center, 2101 E. NASA Parkway, Houston, TX 77058 USA; 5 Centre for Heart, Lung and Vascular Health, School of Health and Exercise Sciences, University of British Columbia Okanagan, Kelowna, BC Canada; 6 Cardiff School of Sport and Health Sciences, Cardiff Metropolitan University, Cardiff, Wales, UK; 7 School of Life Sciences, University of Nevada Las Vegas, 4505 Maryland Parkway, Las Vegas, NV 89154 USA; 8 Department of Ecology and Evolutionary Biology, University of California Santa Cruz, 130 McAllister Way, Santa Cruz, CA 95060 USA; 9 Sea Mammal Research Unit, Scottish Oceans Institute, University of St. Andrews, St Andrews, UK

**Keywords:** Carbon monoxide, Dissociation curve, Hemoglobin, Methemoglobin, Oxygen affinity, Sleep apnea

## Abstract

An exceptionally large blood O_2_ store underlies the remarkable dive performance of elephant seals. However, elevated carboxyhemoglobin (COHb) concentrations in these seals complicate estimations of blood O_2_ content and O_2_ depletion rates, and may also affect monitoring of brain oxygenation in seals with new non-invasive near-infrared (NIR) recorders. Using hemoximetry analyses of blood samples during sleep apneas of juvenile northern elephant seals (*Mirounga angustirostris*), we constructed in vivo Hill plot equations and O_2_–Hb dissociation curves (ODCs). We found: (a) COHb and methemoglobin (both of which do not bind O_2_ and increase hemoglobin (Hb) affinity for O_2_) comprised 8% of Hb, (b) an in vivo P_50_ (partial pressure of O_2_ at 50% Hb saturation, an index of O_2_ affinity of Hb) of 27.1 mm Hg that was Hg 3.4 mm Hg less than that previously determined with an in vitro laboratory approach, and (c) when the in vivo and in vitro Hill plot equations were applied to arterial, hepatic sinus and extradural vein P_O2_ profiles during sleep apneas, the resulting differences in blood O_2_ content and apneic blood O_2_ depletion rates were minor despite higher Hb saturations calculated with the in vivo approach. We conclude that prior blood O_2_ contents and depletion rates determined with the in vitro technique during dives are accurate. The range of arterial Hb saturations calculated from the in vivo and in vitro approaches represents the most realistic Hb saturation data available for evaluation of NIR monitors of arterial Hb saturation in seals.

**Supplementary Information:**

The online version contains supplementary material available at 10.1007/s00360-026-01680-1.

## Introduction

Blood oxygen (O_2_) comprises over 70% of the large total body O_2_ store (96 ml O_2_ kg^− 1^ body mass) of the northern elephant seal (*Mirounga angustirostris*, Gill); the high hemoglobin (Hb) concentration (25 g dl^− 1^) and large blood volume (216 ml kg^− 1^) of this exceptional diver are about 2x and 3x the values in humans, respectively (Ponganis 2015). The contribution of the blood O_2_ store to aerobic metabolism underlies routine dives of 20 to 30 min at sea and breath holds of ~ 20 min of adults during sleep on land (sleep apneas) (Blackwell and Le Boeuf 1993; Le Boeuf et al. 1988; Meir et al. 2009; Robinson et al. 2012; Stockard et al. 2007). To better understand brain oxygenation and metabolism during dives, near-infrared (NIR) spectroscopy techniques have been recently developed and applied to seals to monitor brain oxygenation and measure arterial Hb saturation during diving (McKnight et al. 2019; Ruesch et al. 2021). However, validation of these NIR techniques in seals has not yet been confirmed with blood samples.

Such non-invasive measurements of arterial Hb saturation as well as our understanding of O_2_ transport and depletion during dives are also complicated by the presence of elevated carboxyhemoglobin (COHb) levels in these seals (Tift et al. 2014). Dependent on the design of the NIR monitor, COHb can interfere with accurate assessment of oxygenated Hb (Barker and Badal 2008). Furthermore, not only does carbon monoxide (CO) decrease the functional Hb concentration (COHb does not bind O_2_), CO also increases the O_2_ affinity of Hb (Hb-O_2_ affinity) (Roughton and Darling 1944; Zwart et al. 1984), shifting the O_2_-Hb dissociation curve (ODC) to the left, lowering the P_50_ (partial pressure of O_2_ (P_O2_) at 50% Hb saturation), and potentially increasing Hb saturation at a given P_O2_. Thus, COHb can affect blood O_2_ content in several ways (Hampson 2018; Hlastala et al. 1976; Zijlstra et al. 1996; Dijkhuizen et al. 1977), and, in seals, may have influenced prior estimates of blood O_2_ stores and blood O_2_ depletion during sleep apnea and dives in studies where [COHb]s were not measured (Meir et al. 2009; Stockard et al. 2007).

To evaluate the potential effects of CO and COHb on our prior calculations of blood O_2_ stores and O_2_ depletion in seals, and to develop a protocol for using an arterial Hb saturation profile during sleep apnea to validate NIR-based estimations of arterial Hb saturation in seals, we conducted blood gas and hemoximetric analyses of intermittent blood samples obtained from seals during sleep apnea. These procedures provided data to (a) document COHb, deoxygenated Hb (deoxyHb), oxygenated Hb (oxyHb), total Hb, Hb saturation and P_O2_ and (b) construct in vivo ODCs and estimate P_50_ values from blood that had been analyzed immediately after sampling and in which arterial and venous COHb content had been measured. Then, with the same approach as in Meir et al. (2009), we used these data to convert previously obtained, but unpublished blood P_O2_ profiles recorded during sleep apnea into Hb saturation profiles. Thus, these ODCs, P_50_ data and sleep apnea Hb saturation profiles provided the basis for (a) re-evaluating our earlier constructions of elephant seal ODCs and our prior estimates of blood O_2_ depletion during sleep apnea and diving in seals (Meir et al. 2009), and (b) establishing sleep apnea as a model to evaluate NIR monitors in seals in the future.

Before presenting the experimental approach and study goals, we first provide background for readers on Hb and COHb levels in elephant seals, Hb-O_2_ affinity and its measurement in seals, and on the effects of COHb on blood O_2_ content calculations.

### Carboxyhemoglobin

Remarkably high endogenous carbon monoxide (CO) levels and elevated concentrations of carboxyhemoglobin (COHb) have been documented in elephant seals and in another notable diver, the Weddell seal (*Leptonychotes weddellii*) (Pugh 1959; Tift and Ponganis 2019; Tift et al. 2014). The metabolism of heme during hemoglobin turnover and also during turnover of the elevated myoglobin (Mb) content in muscle (Mb = 7.8 g 100 g^− 1^ muscle (Hassrick et al. 2010) have been considered the primary source of endogenous CO production and the subsequent formation of COHb in these seals (Pugh 1959; Tift et al. 2014).

In adult elephant seals, COHb have been reported to reach 8–10% of total Hb, equivalent to that in human heavy smokers, and more than 4x that in human non-smokers (Tift et al. 2014; Law et al. 1997). Such high levels of COHb complicate estimations of blood O_2_ content and O_2_ depletion in these seals. For example, in adult elephant seals, 10% COHb would decrease the functional Hb concentration (Hb that can bind O_2_) by 2.5 g dl^− 1^ from 25 g dl^− 1^ to 22.5 g dl^− 1^. As for the effect of CO on the Hb-O_2_ affinity, 12-hr cessation of smoking in two-pack-per-day human smokers, decreased COHb from 6.66% to 1.06%, and increased the P_50_ of Hb from 22.9 mm Hg to a near-normal value of 26.4 mm Hg (Kambam et al. 1986).

### Hb–O_2_ affinity

In 2009, Meir and co-workers constructed elephant seal Hb ODCs in the laboratory with the mixing technique (Scheid and Meyer 1978). These results yielded a P_50_ of 30.5 mm Hg at pH 7.40 and allowed examination of the Bohr effect (the shift in O_2_ affinity and P_50_ with changes in blood pH) (Meir et al. 2009). This P_50_ was in the upper range of P_50_ values (24.4 to 31.0 mm Hg) reported in phocid seals, and was similar to one reported earlier in the elephant seal (Lenfant 1969; Lenfant et al. 1969, 1970; Willford et al. 1990; Qvist et al. 1981; Clausen and Ersland 1969; Lapennas and Reeves 1982). The mixing technique is based on the proportional mixing of blood aliquots from blood samples at fixed pH and temperature that had been tonometered with gases (N_2_, O_2_) to produce either 0 or 100% Hb saturation. Proportional mixing of aliquots from the 0 and 100% Hb saturation blood results in samples at different Hb saturations, in which P_O2_ can be measured. This procedure allows estimation of the P_50_ and construction of a Hill plot equation from which an ODC can be constructed. The Hill plot equation is: log [(S_O2_ / (100 - S_O2_)] = (a x log (P_O2_) + b, where S_O2_ = fractional Hb saturation, and a is the cooperativity coefficient (Storz 2016). Meir et al. (2009) also applied the Hill plot equation to intravascular P_O2_ profiles from free-diving, juvenile elephant seals to construct continuous Hb saturation profiles and calculate blood O_2_ depletion during dives.

More recently, a P_50_ of 28.7 mm Hg in juvenile elephant seals was determined from an in vivo ODC constructed with a generalized additive model of hemoximetric data from blood samples collected during sleep apneas (Brown et al. 2024). In contrast to the laboratory approach of Meir et al. the hemoximeter utilizes a spectrophotometric approach to provide Hb saturation data immediately (deoxyHb, oxyHb, COHb, total Hb, Hb saturation, etc.), based on the absorption spectra of these molecules in the blood samples (Juul 2019; Shamir et al. 2012; Zijlstra et al. 1996). This value was 1.8 mm Hg less than that determined with the mixing technique. In addition to differences in computational approach, this small difference may reflect individual variability among seals as well as several other factors. First, different blood gas analyzers were used in the two studies (Brown et al. 2024; Meir et al. 2009). While not significant for clinical decision making, minor differences of 1–3 mm Hg (< 0.5 kPa) in the 40–70 mm Hg P_O2_ range are not unusual between such devices (Bingham et al. 1999; Silverman and Birks 2002; Steinfelder-Visscher et al. 2008; Verwaerde et al. 2002). Second, although COHb is stable during short periods of tonometry (Zijlstra et al. 1996), washout of CO during tonometry of blood samples may decrease the CO and COHb content in the sample and result in a higher P_50_ (Brown et al. 2024; Tift and Ponganis 2019). Third, there may be differences in red blood cell 2,3-diphosphoglycerate (DPG) concentrations between the two procedures. Increased DPG lowers the O_2_-affinity of Hb (Weber 2007; Qvist et al. 1981). If DPG increased during transport to the lab and the tonometry procedure, the ODC would shift to the right increasing the P_50_, potentially accounting for the 1.8 mm Hg higher P_50_ found by Meir et al. (2009). Fourth, unlike pH in the in vitro technique, blood pH is not fixed at a given pH during the in vivo technique. Blood pH may vary among samples throughout the apnea. Brown et al. noted in their review of the human data in their study, the minor but variable differences in pH of in vivo blood samples may contribute to differences in P_50_ compared to values determined with an in vitro technique at a fixed pH (Brown et al. 2024). Given the potential effects of pH, COHb content, and assay techniques on P_50_, the in vivo determination of P_50_ was considered to better reflect Hb-O_2_ affinity under the dynamic physiological conditions of a breath hold (Brown et al. 2024). Balaban and colleagues reached a similar conclusion regarding the value of an in vivo ODC in a study of humans at high altitude (Balaban et al. 2013).

### Blood O_2_ content

Beyond its effect on Hb-O_2_ affinity, elevated COHb also affects the calculation of blood O_2_ content. Typically, and especially in marine mammal O_2_ store calculations, blood O_2_ content is calculated as [Hb] x % Hb saturation x 1.34 ml O_2_ g^− 1^ Hb O_2_ g^− 1^ and adding dissolved O_2_ (0.003 ml O_2_ mm Hg^− 1^ P_O2_) (Zijlstra et al. 1996; Dijkhuizen et al. 1977; Ponganis 2015). Hb saturation is the ratio of oxygenated Hb to the sum of oxygenated Hb and de-oxygenated Hb. The value of 1.34 ml O_2_ g^− 1^ Hb is based in part on the 4:1 molar ratio of O_2_ binding to Hb (Dijkhuizen et al. 1977). For the molecular weight of human Hb, this molar ratio results in 1.39 ml O_2_ g^− 1^ Hb (Dijkhuizen et al. 1977; Dunn et al. 2016; Zijlstra et al. 1996). A value of 1.34 ml O_2_ g^− 1^ Hb is used for calculations when total [Hb] is used in the equation because a small percentage of the total [Hb] is COHb and methemoglobin (metHb), both of which do not bind O_2_ and also increase the Hb-O_2_ affinity (Dijkhuizen et al. 1977). When COHb and metHb data are available, COHb and metHb can be subtracted from the total [Hb] to yield a functional [Hb] (Hb that can bind O_2_). This Hb value should then be multiplied by 1.39 ml O_2_ g^− 1^ Hb and Hb saturation to calculate O_2_ content (Zijlstra et al. 1996). This is especially important in cases of elevated “dyshemoglobins,” which include COHb, metHb, and other variant Hbs.

### Experimental approach and goals

With these findings in mind, we used the approach of Meir et al. (2009) to convert the in vivo hemoximetry data from Brown et al. (2024) into an in vivo Hill plot equation to calculate the P_50_ and to apply this equation to intravascular P_O2_ profiles collected during sleep apnea. We reasoned that use of the same approach to process the P_O2_ and Hb saturation data would allow direct evaluation of the potential effects of COHb on calculations of blood O_2_ depletion during sleep apnea and on prior calculations of blood O_2_ content and blood O_2_ depletion in diving elephant seals.

Elephant seals during sleep apnea were ideal for this investigation because (1) ODCs, Hill plot equations and P_50_s at pHs 7.4, 7.3 and 7.2 had already been determined for elephant seal Hb using the in vitro mixing technique, (2) the molecular weight and absorption spectra of elephant seal Hb were similar to those of humans, (3) hemoximeter data, and pH and blood gas data from intermittent blood sampling during sleep apnea of elephant seals were already available, and (4) continuous arterial, hepatic sinus, and extradural vein P_O2_ and temperature profiles during sleep apnea of juvenile elephant seals were available (Brown et al. 2024; Lincoln et al. 1973; Meir et al. 2009; Stockard et al. 2007; Tift et al. 2014).

Our goals were to (a) assemble an in vivo ODC and Hill plot equation from the available hemoximeter data for each seal and calculate the in vivo P_50_ to compare to the in vitro value previously obtained by Meir et al. (2009), (b) combine the hemoximeter data available for all the seals into a general in vivo Hill plot equation as was done previously for the in vitro data (Meir et al. 2009), (c) apply the general in vivo equation and the general in vitro equation at pH 7.40 to intravascular P_O2_ profiles during sleep apnea to construct Hb saturation and blood O_2_ content profiles throughout the apneas, and (d) examine differences between the two calculations in initial and end-of-apnea Hb saturations and O_2_ contents, and rates of depletion of blood O_2_ during sleep apneas. We hypothesized that (1) the P_50_ calculated with the in vivo equation would be lower than that calculated with the in vitro equation, (2) the largest differences in Hb saturation between the two profiles would depend on the shape of the ODC and location of a given P_O2_ on the ODC, and (3) differences in blood O_2_ content and rate of decline of blood O_2_ calculated with either equation would be minimized because of assumptions in the O_2_ content calculation, i.e., total [Hb], lower Hb saturation and 1.34 ml O_2_ g^− 1^ Hb in the classic in vitro calculation vs. functional [Hb], higher Hb saturation, and 1.39 ml O_2_ g^− 1^ effective Hb in the in vivo calculation (Zijlstra et al. 1996; Zijlstra 2005; Dunn et al. 2016; Dijkhuizen et al. 1977). Because of these differences in assumptions and calculations (see review in Discussion), we expected that blood O_2_ contents and rates of O_2_ depletion calculated with either formula would be similar.

## Methods and materials

### Blood sampling study (conducted in 2022)

Juvenile elephant seals (*n* = 3) were collected and transported from Año Nuevo State Park to Long Marine Laboratory (UC Santa Cruz) in April, 2022 after sedation with 1 mg kg^− 1^ IM tiletamine – zolazepam (Telazol, Aveco Co., Fort Dodge, IA, USA). Anesthesia and catheterization procedures have been previously developed and published (Ponganis et al. 2006a, b). Briefly, approximately four hrs post capture and after re-sedation with half the original dose of Telazol IM, general anesthesia was induced with 5% isoflurane in O_2_ by mask. The seal was intubated, and then maintained on 1-1.5% isoflurane until the procedure was complete. The seal remained on 100% O_2_ until spontaneous ventilation resumed, at which point it was extubated. Spontaneous, long apneas began 4–6 h post anesthesia during which blood sampling was conducted. Approximately 18 h post general anesthesia, the seal was re-sedated with half the original dose of Telazol IV for removal of catheters and probes. After 4–6 h recovery, the seal was returned to the colony. Cefalexin, 1 g IV, was administered every 6 h prophylactically while the seal was catheterized. All procedures were approved by the UC Santa Cruz Chancellor’s Animal Research Committee (Protocol Costd1701) and a National Marine Fisheries marine mammal permit (23188).

Percutaneous catheterizations were performed in the brachial artery:18-g, 23-cm catheter (Arrow, Reading, PA, USA), extradural vein (EDV) in the upper lumbar region with 14-g, 13-cm Angiocath catheter (Becton Dixon, Sandy, UT, USA) and 16-g, 60-cm Mila PICC catheter (Mila International, Huntington Beach, CA, USA), the latter with the catheter tip presumed to be in the cervical region of EDV). Catheters were connected to low-volume extension tubing and stopcocks (Baxter, Deerfield, IL, USA) and maintained with intermittent flushing (normal saline with 1 U ml^− 1^ heparin). Catheters were secured with 2-O nylon suture and a neoprene patch (attached to the fur with Loctite Instant Adhesive 401, Henkel, Dusseldorf, DEU). The arterial line was further secured with application of a Coban wrap around the flipper (VetWrap, 3 M, St. Paul, MN, USA). Apneas were monitored with respiratory fluctuations in EDV pressure (Ponganis et al. 2006a) via an ADInstruments PowerLab data acquisition system and pressure transducer with LabChart software (ADInstruments, Dunedin, NZ).

Paired arterial and venous blood samples were collected in 5-ml heparinized syringes and analyzed using an ABL90 Flex Plus hemoximeter (Radiometer, Copenhagen, DK) (Brown et al. 2024). Sample collection was dictated by the frequency and duration of breath holds, and by the quantity of the hemoximeter stock solution available. Most samples (78%) were collected during apneas. In addition to blood gases, blood pH, and [lactate], the hemoximeter provided total [Hb] (g dl^− 1^), Hb saturation (oxyHb / (oxyHb + deoxyHb), dexoxyHb (%), oxy Hb (%), CoHb (%) and metHb (%).

Total [Hb], CoHb, metHb, pH, and P_CO2_ were organized into three categories: all samples combined, arterial samples, and venous samples. This grouping enabled analysis within individual seals as well as comparisons between arterial and venous samples.

The hemoximeter data were assembled into different groups for P_50_ analysis: combined arterial and venous data of each seal, arterial data alone of each seal, venous data alone of each seal, arterial data of all seals pooled, venous data of all seals pooled, combined arterial and venous data of all seals pooled, and all data of all seals for Hb saturations between 30 and 80%. Hill plot equations for each data set category were constructed from the P_O2_ and Hb saturation data from the hemoximeter with linear regression in Origin (Northampton, MA USA). The Hill equation for a given data set was then used to calculate Hb saturations from the P_O2_s measured with the hemoximeter. ODCs were constructed in Origin (logistic regression) from these P_O2_ data and the corresponding calculated Hb saturation data. The P_50_ of each data set category was determined graphically as the P_O2_ at 50% Hb saturation on the ODC. It was expected that the combined arterial and venous data results would be most similar to the approach used by Meir et al. (2009) to construct Hb saturation profiles from P_O2_ profiles based on all available data. We also determined the P_50_ using only samples in the 30 to 80% saturation range based on the recommendation to use data in this Hb saturation range (the linear segment of the ODC) when calculating P_50_ in human data (Severinghaus 1979).

Calculation of blood O_2_ content assumed a total [Hb] of 25 g dl^− 1^ in order to compare the in vivo and in vitro approaches. The 25 g dl^− 1^ value, typical of elephant seals (Ponganis 2015), was determined using a cyanmethemoglobin technique as part of the Meir et al. (2009) study Blood O_2_ contents calculated with the in vivo approach (this study) assumed a functional [Hb] (i.e., Hb that can bind O_2_) in which COHb and metHb were subtracted from the total Hb (= total [Hb] x (1-((COHb + metHb)/100), a Hb saturation based on an ODC and Hill plot equation constructed from the combined hemoximetric data from the three seals, an O_2_ binding capacity of 1.39 ml O_2_ g^− 1^ Hb (Zijlstra et al. 1996), and a dissolved O_2_ capacity of blood of 0.003 ml O_2_ dl^− 1^ mm Hg^− 1^ P_O2_ as in Meir et al. (2009). Blood O_2_ contents calculated with the in vitro approach (Meir et al. 2009) utilized a total [Hb] of 25 g dl^− 1^, Hb saturations calculated in 2009 from an ODC and Hill equation determined in the laboratory with a gas-mixing technique, an O_2_ binding capacity of 1.34 ml O_2_ g^− 1^ Hb, and a dissolved O_2_ capacity of blood of 0.003 ml O_2_ dl^− 1^ mm Hg^− 1^ P_O2_.

### P_O2_ profile study (data collected in 2006–2008 (Meir et al. 2009)

The sleep apnea P_O2_ profiles were collected from juvenile elephant seals in Spring 2006–2008 as part of a study by Meir et al. (2009) that investigated P_O2_ dynamics and blood O_2_ depletion during diving. Detailed methods are available in Meir et al. (2009), but briefly, anesthesia and catheterization techniques were similar to the current blood sampling study described above. However, in Meir et al. (2009), an intravascular P_O2_ electrode and thermistor were placed in one of three sites, the aorta, hepatic sinus (HS) or the EDV and connected to a data logger with each of the seals instrumented at a different site. As in the current study, four to six hrs after anesthesia, the seals exhibited long breath holds overnight; they were released to sea the next morning for the diving study. A temporary EDV catheter, removed prior to release, allowed monitoring of respiratory oscillations in EDV pressure to identify sleep apneas. The EDV pressure was transduced with a Hewlett-Packard blood pressure transducer system (H-P 78304 A/78205 D), on a Dell Dimension 8100 personal computer with AcqKnowledge software and a Biopac MP100 System interface (Santa Barbara, CA, USA) (Ponganis et al. 2006a). Data loggers and P_O2_ and temperature probes were removed after the seals returned to the colony (IM Telazol sedation as described above for capture). Elephant seal Hb ODCs were determined in the laboratory with the mixing technique (Scheid and Meyer 1978). All procedures were approved by the UCSC Chancellor’s Animal Research Committee and a National Marine Fisheries marine mammal permit (87-1743-02).

Previously unpublished aortic, HS and EDV P_O2_ and temperature profiles from Meir et al. (2009) were identified in three seals during serial episodes of sleep apnea that were documented by eupneic respiratory oscillations in simultaneous EDV pressure profiles (Ponganis et al. 2006a). From these P_O2_ profiles, Hb saturation and blood O_2_ content profiles were constructed with both the in vitro and in vivo approaches. Apneic blood O_2_ depletion rates were calculated from both the in vitro and in vivo approaches using start-of-apnea and end-of-apnea blood O_2_ contents. A total [Hb] of 25 g dl^− 1^, the average value of the seals in Meir et al. (2009) was used in the in vitro calculation. The in vivo calculation used the functional [Hb] that was based on that average value and the percentage COHb and metHb in the current study (see above description).

### Statistics

To determine if blood parameters important in blood oxygen calculations differed between paired venous and arterial samples, we performed linear mixed-effects models (Cran R, package nlme). Sample type (venous or arterial) was the fixed effect, and to account for the lack of independence, sample pair (venous and arterial sample collected simultaneously) nested in seal ID was included as the random effect. The response variable was the blood parameter of interest (COHb, metHb, pH, and P_CO2_). We also performed a linear mixed-effect model to compare depletion rates calculated using the in vitro and the in vivo-derived equations. The fixed effects were equation (in vitro vs. in vivo), probe location (aorta, HS, or EDV), and the interaction. Sample pair was included as a random effect. Seal was not included as a random effect because there was only one seal per probe location. Because the interaction term was significant, indicating that the impact of equation on depletion rate differed among the three locations, we ran a separate mixed-effect model for each location with equation as a fixed effect, and sample pair as the random effect. Model assumptions were confirmed by examination of residual plots (Zuur et al. 2009).

## Results

### Arterial versus venous sample data

In the hemoximetry study, mean [Hb] in the three yearling seals (144–155 kg, *n* = 27 paired arterial/venous samples) ranged from 21.9 to 23.4 g dl^− 1^ (Table 1). Similarly, mean COHb and metHb levels for each seal varied slightly between 4.8 and 6.7% and 2.1 to 2.3%, respectively (Table 1). In each seal, mean arterial and venous COHb, and mean arterial and venous metHb differed by less than 0.5% and 0.2%, respectively (Table S1). Blood pH values fell within a narrow range between 7.36 and 7.39 (Table 1).

**Table 1:** Seal characteristics, hemoglobin (Hb) parameters and blood pH of three sleeping elephant seals in 2022

Seal ID, gender, age	Body Mass	[Hb]	COHb	metHb	COHb + metHb	Blood pH
	kg	g dl^− 1^	%	%	%	pH units
Seal 1, F, yearling	166	23.4 (0.14)	5.9 (0.05)	2.1 (0.10)	8.0 (0.11)	7.39 (0.003)
Seal 3, F, yearling	144	22.2 (0.17)	4.8 (0.06)	2.2 (0.05)	7.0 (0.10)	7.37 (0.002)
Seal 5, M, yearling	161	21.9 (0.14)	6.7 (0.09)	2.3 (0.04)	9.0 (0.12)	7.36 (0.005)

Arterial and venous data, collected during both apnea and eupnea and measured with the ABL90 hemoximeter were pooled for each seal (*n* = 28, 16 and 10 for seals 1, 3 and 5, respectively). These parameters affect both the oxygen affinity of Hb and the quantity of effective Hb (Hb that can bind oxygen). Data are expressed as mean (SE)

*ID* identification, *[Hb]* total Hb concentration, *COHb* carboxyhemoglobin, *metHb* methemoglobin, *SE* standard error

Although COHb, pH, and P_CO2_ differed significantly between paired arterial and venous samples across all seals, the differences were minimal (Table 2A). Arterial COHb and pH were 0.22% and 0.02 units higher, respectively, while arterial P_CO2_ was 2.5 mm Hg lower (Table 2A). No significant difference was observed between arterial and venous metHb (Table 2A).

**Table 2:** Mixed effects-model results comparing (A) paired arterial and venous parameters (COHb, metHb, pH and P_CO2_ from all seals) that affect the O_2_ affinity of hemoglobin (Hb) and the P_50_ (partial pressure of O_2_ at 50% Hb saturation) and (B) blood O_2_ depletion rates calculated using the in vitro vs. in vivo approaches (see text)

(A) Arterial and venous parameter comparison							
Parameter	Arterial	Venous	Difference	t_26_	*p*	icc	*R*^2^_marg_ / *R*^2^_cond_
**COHb** (%)	5.81 ± 0.14	5.59 ± 0.14	-0.22 ± 0.05	-4.469	< 0.001	0.438	0.013 / 0.966
metHb (%)	2.12 ± 0.11	2.22 ± 0.02	0.09 ± 0.10	0.920	0.366	0.118	0.014 / 0.140
**pH **(pH units)	7.39 ± 0.004	7.37 ± 0.003	-0.02 ± 0.002	-7.266	< 0.001	0.474	0.161 / 0.839
**P**_**CO2**_ (mm Hg)	55.32 ± 0.93	57.84 ± 0.59	2.52 ± 0.06	4.191	< 0.001	0.471	0.076 / 0.770

(B) In vitro versus in vivo approach comparison – Blood O_**2**_ depletion rate (ml O_2_ dl^−1^min^− 1^)							
All locations combined (Fixed effects: Equation, electrode location, and interaction)							
	In vitro	In vivo	Difference	F	p	icc	R^2^_marg_ / R^2^_cond_
Equation	2.47 ± 0.07	2.44 ± 0.09	-0.02 ± 0.04	F_1,28_= 3.12	0.09	0.279	0.000 / 0.934
**Location**				F_2,28_ = 98.98	< 0.001		
Interaction				F_2,28_ = 137.66	< 0.001		
Locations separated (Fixed effect: Equation)							
**Equation – Art.**	1.84 ± 0.04	1.40 ± 0.05	-0.44 ± 0.02	F_1,4_ = 634.71	< 0.001	0.916	0.855 / 0.988
Equation - HS	2.61 ± 0.04	2.56 ± 0.04	-0.05 ± 0.03	F_1,11_ = 3.46	0.09	0.810	0.028 / 0.815
**Equation - EDV**	2.57 ± 0.05	2.72 ± 0.04	0.15 ± 0.01	F_1,13_ = 119.79	< 0.001	0.951	0.180 / 0.960

Values are mean ± SE. Values that are significantly different are in bold. Marginal R^2^ indicates the amount of variance accounted for by fixed variables. Conditional R2 indicates the amount of variance accounted for by the entire model. icc is the intra-correlation coefficient that indicates the amount of variance attributed to the random effects

*P*_*CO2*_ partial pressure of carbon dioxide, *COHb* carboxyhemoglobin, *metHb* methemoglobin

### In vivo O_2_ dissociation curve

**Fig. 1:**
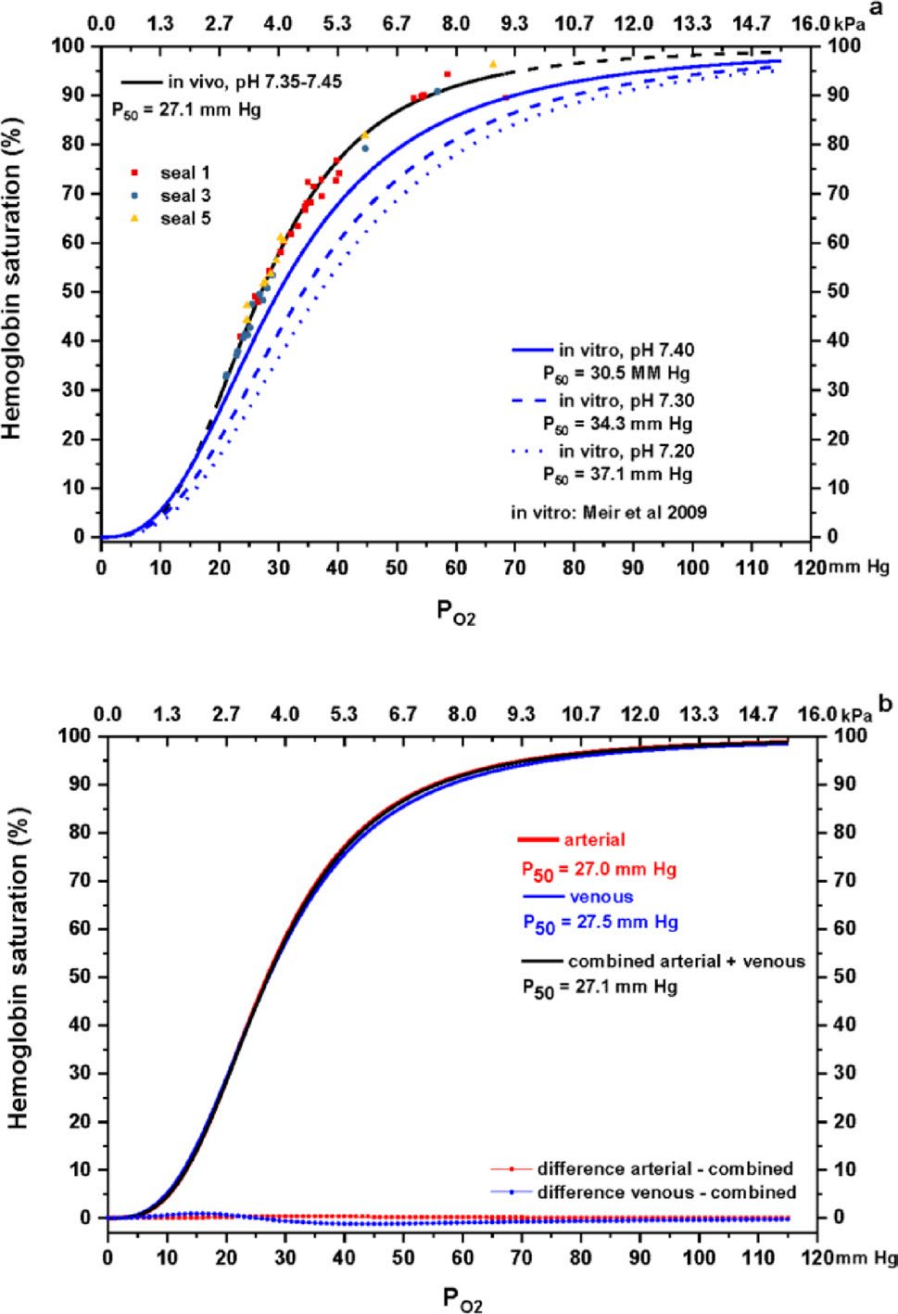
O_2_-Hb dissociation curve (ODC) of the elephant seal. **a** The in vivo ODC is constructed from blood gas analyses and Hb saturations determined on an ABL 90 hemoximeter (solid black line -range of data, dashed line extrapolation of formula). The in vitro mixing technique ODC (blue) was determined at pH 7.40, 7.30 and 7.20 with tonometry of blood in the laboratory (Meir et al. 2009). **b** Comparison of the in vivo ODC created with arterial (red) (*n* = 27) or venous (blue) samples (*n* = 27) from 3 seals. The black curve is all samples combined. There is minimal difference between the arterial or venous ODC’s when compared to the combined (dashed red and blue lines)

The mean P_50_ calculated from the ODC constructed from pooled samples across all three seals (*n* = 54) was 27.1 mm Hg (Table 3; Fig. 1a). When restricted to samples within the 30%-80% Hb saturation range (*n* = 46), the mean was 27.2 mm Hg (Table 3). Arterial and venous P_50_s for each seal ranged from 26.8 to 27.1 mm Hg and 27.1 to 27.9 mm Hg, respectively. When arterial and venous data were each pooled across all seals, the resulting arterial P_50_ was 27.0 mm Hg, while the venous P_50_ was slightly higher at 27.5 mm Hg (Table S1).

**Table 3:** Hill plot equations (log [(S_O2_ / (100 - S_O2_)] = (a x log (P_O2_) + b) and calculated P50s resulting from the in vitro mixing technique used by Meir et al. (2009), and from the in vivo hemoglobin saturation (S_O2_) and P_O2_ data in this study, including: all available data from 3 elephant seals, 30% to 80% saturation data from 3 seals, and data from the individual seals

	a	b	*r* ^2^	*P*_50_ (mm Hg)	*n*
In vitro, pH 7.4 (from Meir et al. 2009)	2.60211	− 3.84507	0.99	30.5	61
In vivo (all data from 3 seals)	3.05428 (0.07045)	– 4.37508 (0.10645)	0.97	27.1	54
In vivo (30–80% saturations from 3 seals)	2.88063 (0.05774)	– 4.12544 (0.08458)	0.98	27.2	46
In vivo seal 1	2.93780 (0.12387)	-4.20090 (0.19249)	0.95	27.1	28
In vivo seal 3	2.96367 (0.06241)	-4.25816 (0.08953)	0.99	27.3	16
In vivo seal 5	3.39736 (0.1362)	-4.85516 (0.20548)	0.99	27.1	10

Slope (a) and intercept (b) include (SE). All regressions were significant (*p* < 0.05)

The in vivo ODC, determined from the combined arterial and venous data of all seals, closely overlapped with the separately determined in vivo arterial and venous ODCs (Fig. 1b). The maximum difference in saturation between the arterial and combined ODCs was 0.39% saturation at 31 mm Hg while the maximum difference between the venous and combined ODCs was 0.92% saturation at 15 mm Hg (Fig. 1b).

### In vivo and in vitro O_2_ dissociation curve comparison

**Fig. 2:**
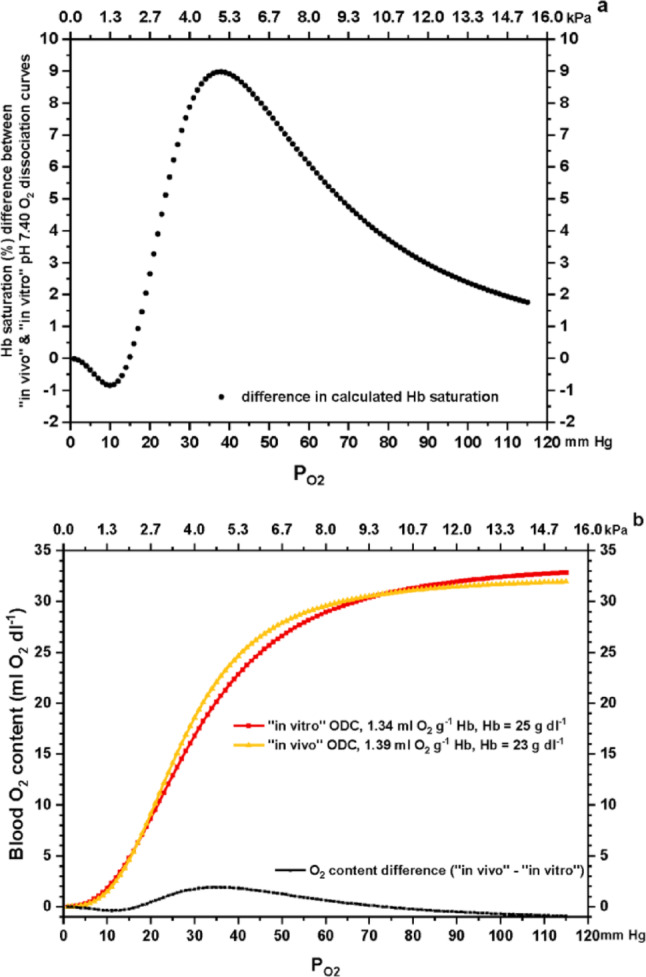
Difference in Hb saturation and blood O_2_ content calculated with the in vivo O_2_ dissociation curve and the Meir pH 7.4 (mixing technique) O_2_ dissociation curve. **a** There is almost a 10% difference in saturation for P_O2_s of 35–40 mm Hg. O_2_ affinity becomes marginally lower at P_O2_s < 20 mm Hg in the mixing technique dissociation curve but this may be due to the general inaccuracy of O_2_ dissociation curves at such low P_O2_ and saturation values (Severinghaus 1979). The higher affinity of the in vivo dissociation curve may be secondary to differences in analytical techniques or to differences in concentration of carboxyhemoglobin or 2,3-diphosphoglycerate in the blood samples secondary to the analytic approach. **b** Difference in O_2_ content calculated with the in vitro and in vivo formulae versus P_O2_. The in vivo formula uses an effective Hb of 23 g dl^− 1^ (based on 8% COHb and metHb) versus 25 g dl^− 1^ for the in vitro calculation. The in vivo formula also has a higher O_2_ binding capacity (1.39 ml O_2_ g^− 1^ Hb) than in the in vitro calculation (1.34 ml O_2_ per g^− 1^ Hb). A total [Hb] of 25 g dl^− 1^ was assumed in the calculation (Meir et al. 2009)

Given the similarity between the arterial and venous curves, the in vivo ODC determined from the pooled arterial and venous data was considered most appropriate for comparison to the previously published in vitro ODC at pH 7.40 (Meir et al. 2009). Differences in Hb saturations between the in vivo and in vitro ODCs varied with P_O2_ – with smaller differences at low and high P_O2_s (Figs. 1a and 2a). The largest difference of 9% saturation was at a P_O2_ of 38 mm Hg, while the difference decreased to 2% saturation at P_O2_ values 19 mm Hg and 107 mm Hg. Corresponding differences in blood O_2_ content calculated with the in vitro and in vivo techniques for a [Hb] of 25 g Hb dl^− 1^ peaked at approximately 1.9 ml O_2_ dl^− 1^ at P_O2_s of 32–38 mm Hg, and decreased to 0.5 ml O_2_ dl^− 1^ at 20 mm Hg and 63 mm Hg (Fig. 2b).

### Hemoglobin saturation profiles during apnea and eupnea

We constructed and compared Hb saturation profiles from P_O2_ profiles using both the published in vitro ODC and the in vivo ODC reported above in three sleeping elephant seals (180 kg male, 160 kg female, 160 kg female) with intravascular P_O2_ electrodes placed in the aorta, HS, or EDV, respectively. The P_O2_ profiles during sleep apnea and eupnea, identified from eupneic pressure oscillations in the EDV, were consistent in pattern and in maximum and minimum values at each of the three sites (Fig. S1). Temperature fluctuations within each apnea-eupnea cycle were minimal, remaining within 0.5 °C(Fig. S1). Over the course of recordings, aortic temperature ranged between 37.1 and 37.5 °C over 70 min; HS temperature from 36.7 to 37.2 °C over 150 min, and EDV temperature from 36.5 to 37.1 °C over 170 min. Mean apnea durations for seals with the O_2_ electrode in the aorta, HS, and EDV were 5.2, 6.6, and 5.8 min, respectively (Table 4).

**Fig. 3:**
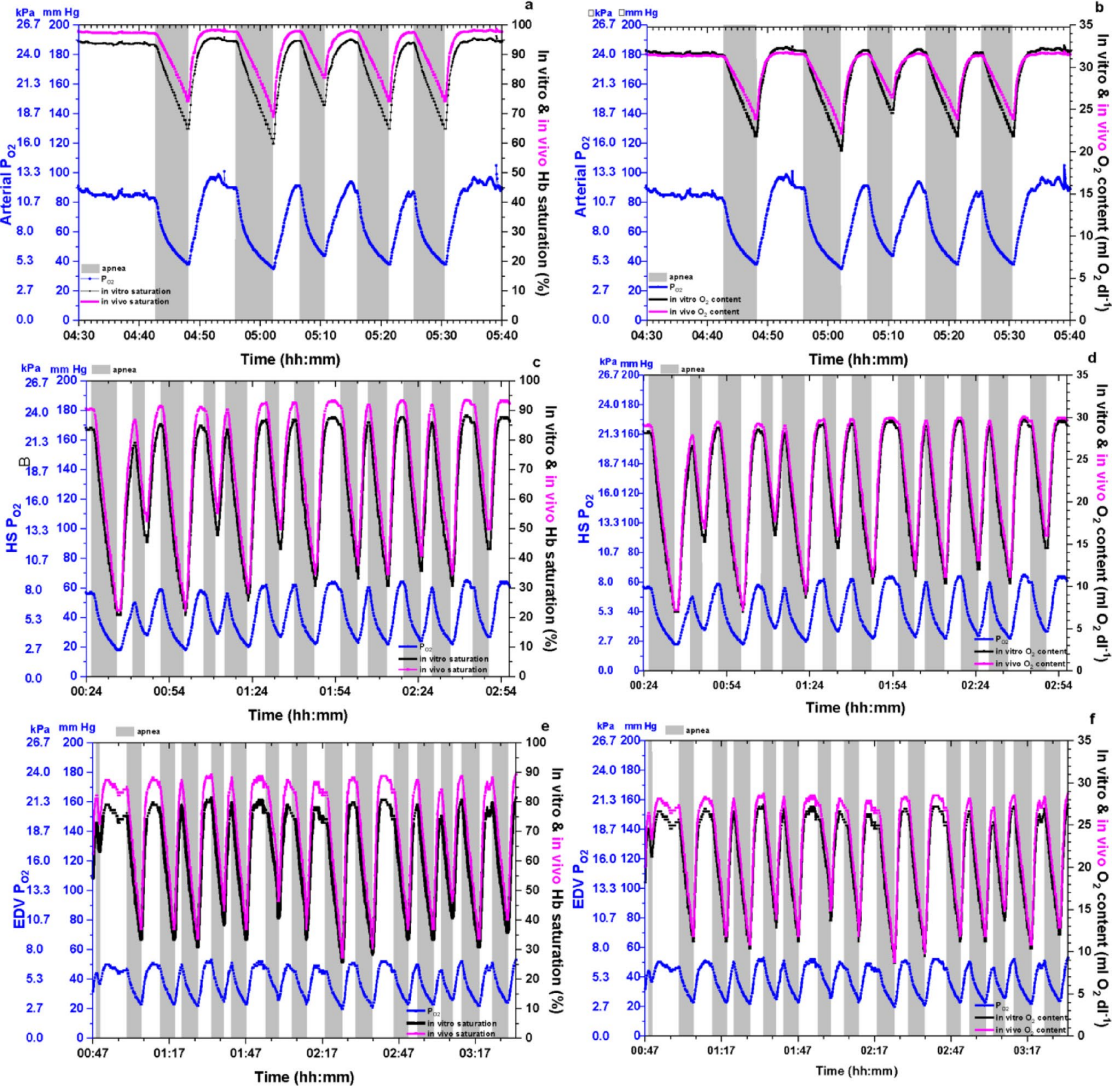
Arterial, hepatic sinus (HS) and extradural vein (EDV) P_O2_, hemoglobin (Hb) saturation and O_**2**_ content profiles during sleep apneas. Hb saturation and O_2_ contents were calculated with both an in vitro and in vivo approach (see text and Fig. 2 legend). **a**, **b** Arterial. **c**, **d** Hepatic sinus. **e**, **f** Extradural vein

We constructed Hb saturation profiles from the P_O2_ profiles in the aorta (five apneas over 1 h), HS (12 apneas over 2.6 h), and EDV (15 apneas over 2.8 h) using both the in vivo and in vitro Hill plot equations (Fig. 3a, b). The in vivo Hill plot equation resulted in 3% to 8% higher initial and final mean saturations at each site (Table 4). Corresponding differences in calculated blood O_2_ contents were ≤ 1 ml O_2_ dl^− 1^, except for final arterial saturation which showed a larger difference of 1.9 ml O_2_ dl^− 1^ (Table 4).

**Table 4:** Comparison of blood O_2_ indices calculated by application of either the in vitro O_2_–Hb dissociation curve (ODC) or the in vivo ODC to continuous P_O2_ profiles during sleep apneas of three different elephant seals, each with a P_O2_ electrode in either the aorta, hepatic sinus (HS), or extradural vein (EDV)

	Duration	*P*_O2_ initial	*P*_O2_ final	Sat initial	Sat final	O_2_ content initial	O_2_ content final	Blood O_2_ depletion rate
	min	mm Hg	mm Hg	%	%	ml O_2_ dl^− 1^	ml O_2_ dl^− 1^	ml O_2_ dl^−1^min^− 1^
Aorta								
In vitro	5.23 (0.30)	87.2 (1.36)	38.8 (1.46)	94.1 (0.22)	65.8 (4.25)	31.8 (0.09)	22.1 (0.04)	1.8 (0.04)
In vivo				97.3 (0.13)	74.6 (4.10)	31.4 (0.05)	24.0 (0.66)	1.4 (0.05)
HS								
In vitro	6.56 (0.43)	58.3 (0.99)	22.7 (1.02)	84.7 (0.63)	34.2 (2.73)	28.5 (0.21)	11.5 (0.92)	2.6 (0.04)
In vivo				91.1 (0.47)	38.8 (3.36)	29.3 (0.16)	12.5 (1.08)	2.6 (0.04)
EDV								
In vitro	5.80 (0.23)	50.6 (2.00)	23.1 (0.46)	78.2 (0.77)	33.6 (1.13)	26.1 (0.22)	11.3 (0.38)	2.6 (0.05)
In vivo				86.1 (0.55)	37.4 (1.45)	27.7 (0.17)	12.0 (0.47)	2.7 (0.04)

Mean arterial blood O_2_ depletion rate calculated with the in vivo ODC was 78% of that calculated with the in vitro ODC. Venous blood O_2_ depletion rates calculated with the two ODCs were indistinguishable in both the HS and EDV. A [Hb] of 25 g dl^− 1^ was assumed in the O_2_ content estimate (Meir et al. 2009). Number of apneas in each category: aorta, 5, HS, 12, and EDV, 14. See text for review of calculations. Data are expressed as mean (SE)

*Hb* hemoglobin, *P*_O2_ partial pressure of O_2_, *Sat* hemoglobin saturation

A mixed-effects model examining apneic blood O_2_ depletion rate, including both equation type and sampling location, revealed that location significantly influenced how comparable the calculations were, prompting separate analysis by electrode site. In the HS, depletion rates did not differ between the in vivo and in vitro calculations (2.6 ml O_2_ dl^− 1^ min^− 1^, *p* = 0.09, Table 2B). EDV rates, although significantly different, were nearly identical (2.6 in vitro vs. 2.7 in vivo ml O_2_ dl^− 1^ min^− 1^, *p* < 0.001, Table 2B). However, in the aorta, depletion rates calculated using the in vivo equation were 22% slower than those from the in vitro Eq. (1.4 versus 1.8 ml O_2_ dl^− 1^ min^− 1^, *p* < 0.001, Tables 2B, 4).

## Discussion

### Blood parameters influencing Hb–O_2_ affinity and the binding of O_2_ to Hb

Hemoglobin concentrations and COHb levels from all blood samples in each of the three elephant seals in the hemoximetry study (Table 1) were typical of values previously reported in yearlings (Thorson and Le Boeuf 1994; Tift et al. 2014). As far as we know, metHb levels have not been reported in pinnipeds, but the values in the seals were in the reported normal human ranges of < 1% to 3% (Kaminecki and Huang 2021; Cortazzo and Lichtman 2014; Wright et al. 1999). In these seals, 8% of the total Hb content was either bound to CO (COHb) or oxidized to Fe^3+^ (metHb), and, therefore, unavailable for O_2_ transport (Table 1).

Although some small but statistically significant differences were observed in paired arterial and venous data for COHb, pH and P_CO2_ (Tables 1 and 2A, S1), these differences were minor and well within the range of typical physiological variation (0.22% COHb, 0.02 pH units, and 2.5 mm Hg P_CO2_, Table 2A). This was expected for several reasons. First, human arterial and venous COHb levels were not significantly different in cases of CO toxicity (Touger et al. 1995). Second, blood pH was well-buffered in pinnipeds and changed minimally during sleep apnea of elephant seals (Stockard et al. 2007). Lastly, most blood samples (78%) were collected during apnea, and arterial and venous P_CO2_ were typically in the same range during sleep apnea of elephant seals (Stockard et al. 2007). Taken together, the similarities in these paired arterial-venous data support using a pooled data set to determine a general Hill plot equation and P_50_ that can be applied to intravascular P_O2_ profiles to construct continuous Hb saturation profiles during breath holds.

### Hill plot equations and P_50_s

To determine the appropriateness of using a generalized Hill plot equation based on pooled arterial and venous data from all the seals, Hill plot equations were constructed from arterial and venous data separately for the individual seals and for all the seals combined (Table S1). As expected from the COHb, metHb, and pH findings reviewed above, the P_50_s determined from the resulting ODCs were all similar, near 27 mm Hg (3.6 kPa, Table S1). Therefore, arterial and venous data were combined to construct Hill plot equations and calculate P_50_s for the individual seals and for all the seals combined. The P_50_ calculated from all the seals (27.1 mm Hg, 3.6 kPa) was identical to those calculated for two of the individual seals, and 0.2 mm Hg less than that of seal 3 (Table 2). Based on the recommendation that P_50_ be determined on the most linear portion of ODC (Severinghaus 1979), we also constructed a Hill plot based on the 30–80% combined data for all the seals, and again obtained a similar P_50_ of 27.2 mm Hg. Therefore, we conclude that the use of an in vivo Hill plot equation and ODC, based on the combined arterial and venous data of all the seals (P_50_ 27.1 mm Hg), is appropriate for comparison to the in vitro, pH 7.4 ODC and for the conversion of P_O2_ profiles of other elephant seals to Hb saturation profiles.

### Comparison of the in vivo and in vitro Hill plot equations, O_2_–Hb dissociation curves and P_50_s

The in vivo ODC of the elephant seal was left-shifted compared to the in vitro ODC (pH 7.40) with a P_50_ that was 3.4 mm Hg (0.45 kPa) lower (Fig. 1). Blood pH was an unlikely driver of this shift because pH of the blood samples was similar to the 7.40 value in the in vitro technique, ranging between 7.35 and 7.45, with mean values between pH 7.36 and 7.39 in each seal (Table 1). Blood temperature was also an unlikely cause of this difference because temperature during the in vitro technique was 37 °C (Meir et al. 2009) and during sleep apnea, aortic, hepatic sinus and extradural vein temperatures ranged from 36.5 to 37.5 °C (Fig. S1), similar to pulmonary artery temperatures reported previously during sleep apnea (Ponganis et al. 2006b). The difference between the in vivo and in vitro ODCs may be secondary to differences in technique (i.e., analyzer used, sample stability during transport and tonometry prior to measurement in the mixing technique (red blood cell DPG level), possible washout of CO during the tonometry). Error in the accuracy of volumetric mixing or in the measurement of oxyHb and deoxyHb with the hemoximeter are unlikely given the successful validation of P_50_s of other species in the mixing technique study and because peaks in the absorbance spectra of elephant seal Hb match those of Hb in other mammalian species, including humans (Meir et al. 2009; Tift et al. 2014). Further investigation is needed to identify the cause of the difference between the ODCs and P_50_s determined with the in vitro and in vivo Hill plot equations. We assume the 1.7 mm Hg difference in P_50_s determined in this study (27.1 mm Hg) and in the study of Brown et al. (2024) (28.8 mm Hg) on the same blood samples is secondary to differences in computational technique (Hill plot equations versus general additive models).

Applying the in vivo Hill plot equation (determined from all blood samples) produced higher Hb saturations than those calculated with the published in vitro Hill equation across a range of P_O2_s from 1 to 115 mm Hg (Fig. 2a) except at P_O2_s < 15 mm Hg, where the in vivo saturation transiently decreased ~ 1% relative to the in vitro value (Fig. 2a). This small difference likely reflects limited data and decreased accuracy of the equation at extremely low saturations (Severinghaus 1979). Additional data at extremely low P_O2_ and saturations would better define the shape of the ODC and determine whether the elephant seal ODC becomes more hyperbolic as observed in human blood in the presence of CO (Roughton and Darling 1944). In the elephant seal, the largest saturation difference between the two equations was 9% at a P_O2_ of 38 mm Hg, tapering to 2% saturation difference by 19 mm Hg and 107 mm Hg (Fig. 2a). Despite the lower effective Hb concentration used in the in vivo blood O_2_ content calculation, the increased affinity and saturation of Hb in the in vivo formula resulted in blood O_2_ contents that were equivalent to or greater than those calculated with the in vitro approach (Fig. 2b). The largest differences were ~ 1.9 ml O_2_ dl^− 1^ at P_O2_s of 32–38 mm Hg, decreasing to 0.5 ml O_2_ dl^− 1^ at 20 mm Hg and 63 mm Hg (Fig. 2b). If the higher O_2_ content calculated with the in vivo approach is secondary to CO washout during tonometry in the in vitro approach, this finding is similar to that found by Roughton and Darling (1944) in human blood. In that study, at a P_O2_ of 20 mm Hg, blood with 20% COHb had a blood O_2_ content about 0.8 ml O_2_ dl^− 1^ greater than that in blood in the absence of CO.

Based on these results, we applied our in vivo Hill plot equation determined from all samples in the three yearling seals of this study, and the published in vitro pH 7.40 Hill equation to intravascular P_O2_ profiles during sleep apnea that were obtained from yearling elephant seals in the prior study (Meir et al. 2009). This allowed construction of Hb saturation profiles from the two different Hill plot equations and comparison of Hb saturations, blood O_2_ contents, and apneic blood O_2_ depletion rates calculated with the two different equations.

### Sleep apnea P_O2_ and temperature profiles

Sleep apnea intravascular P_O2_ and temperature profiles (Fig. S1) were ideal for applying the in vivo Hill plot equation to construct Hb saturation profiles because the P_O2_ profiles and in vivo blood sample data were collected under comparable conditions in the same age group of seals. In addition, temperature fluctuations during apnea-eupnea cycles were minimal, thus limiting any effect of change in temperature on Hb-O_2_ affinity and Hb saturation. The seals in both studies were in the same size range (140–180 kg), but the translocation seals had a higher [Hb], 25 g dl^− 1^ (Meir et al. 2009). That higher [Hb] was used for both the in vivo and in vitro approaches in blood O_2_ content calculations with those seals. Although hematocrit and [Hb] increased variably during sleep apnea (Castellini et al. 1986; Stockard et al. 2007), in the absence of serial blood sampling in the seals equipped with the P_O2_ electrode, [Hb] was assumed constant during calculations of O_2_ content profiles.

### Sleep apnea Hb saturation profiles, blood O_2_ content profiles, and apneic blood O_2_ depletion rates

Hb saturation profiles during sleep apnea reconstructed with the in vivo equation were consistently higher than those calculated with the in vitro equation (Fig. 3a). In the aorta, the difference in start-of-apnea mean saturation was 3.2% saturation, in the HS, 6.4% saturation, and in the EDV, 7.9% saturation (Table 3). The difference in mean end-of-apnea saturation was 8.8% saturation in the aorta, 4.6% saturation in the HS, and 1.6% saturation in the EDV. As had been described in Fig. 2a, these differences were a function of the shape of ODC used, the P_O2_ value at the start or end of an apnea, and the location of that P_O2_ value on a given ODC.

Based on these Hb saturation profiles, blood O_2_ content profiles for these sleep apneas were constructed with calculations specific to each approach (in vivo vs. in vitro, Fig. 2b). The in vivo approach used a functional [Hb] of 23 g dl^− 1^ (total [Hb] x 0.92 due to 8% (COHb + metHb) content) and an O_2_ binding capacity of 1.39 ml O_2_ g^− 1^ Hb. The in vitro approach utilized a total [Hb] of 25 g dl^− 1^ and 1.34 ml O_2_ g^− 1^ Hb (Meir et al. 2009).

Despite differences in initial and final mean Hb saturations as large as 9% saturation between methods during the apnea, the differences in mean blood O_2_ contents were small, ≤ 1 ml O_2_ dl^− 1^ (Table 4). Even with the lower functional [Hb] used in the in vivo approach, blood O_2_ contents at all electrode sites were greater than that calculated with the in vitro approach except for the initial arterial value (Table 4). These results are due to the differences in O_2_ binding capacities in the two formulae, to differences in the ODCs and to the position of a given P_O2_ value on the ODC.

Using calculated O_2_ content values from both the in vivo and in vitro equations, we determined blood O_2_ depletion rates for the aorta, HS, and EDV. The two equations produced different arterial blood O_2_ depletion rates, with the in vivo rate being 0.4 ml O_2_ dl^− 1^ min^− 1^ slower than the in vitro equation estimate (Tables 2 and 4). In contrast, differences between venous depletion rates estimated from the two equations were minimal. There was no difference between the two methods in the HS depletion rates, while in the EDV, the in vivo depletion rate was slightly higher (0.15 ml O_2_ dl^− 1^ min^− 1^) than the in vitro rate (Tables 2 and 4). Overall, O_2_ depletion rates at the two venous sites were notably similar. The decrease in the effective [Hb] in the in vivo approach, due to the presence of COHb and metHb, appeared to be compensated by the increase in Hb-O_2_ affinity and higher start-of-apnea Hb saturations.

The apneic arterial and venous blood O_2_ depletion rates calculated with either approach during these apneas were about one-third less than arterial values and 30% greater than venous values previously reported during sleep apnea (Stockard et al. 2007). However, those earlier estimates were based on regressions of blood sample data from multiple seals from a rehabilitation program that were substantially smaller (~ 1/3 the body mass) and younger than the translocated seals. Consequently, we consider the values calculated from P_O2_ profiles with either the in vitro or in vivo technique to be more realistic for these translocated seals than the apneic depletion rates reported in the earlier study (Stockard et al. 2007).

The overall similarity between O_2_ depletion rates calculated with the in vivo and in vitro techniques during sleep apnea reassures to us that O_2_ depletion rates during dives, calculated with the in vitro technique, still remain reasonable estimates for a diving elephant seal (Meir et al. 2009). Although sleep apnea and diving differ in important ways, the comparison is still useful. In elephant seals, dives were considerably longer and heart rates were slower than during sleep apneas (Andrews et al. 1997; Blackwell and Le Boeuf 1993). Maximum and minimum arterial and venous P_O2_s during dives were typically higher and lower, respectively, than those during sleep apnea in this study (Meir et al. 2009). At such values during dives, differences between in vitro and in vivo calculations of maximum and minimum blood O_2_ contents were minimal (Fig. 2b). Blood pH, P_CO2_, and the in vivo P_50_ probably also differ between sleep apnea and diving. We conclude that the near equivalence of the two techniques to calculate blood oxygen content during sleep apnea supports the prior estimates of blood O_2_ depletion rates determined with the in vitro approach in diving seals.

Together, the in vivo and in vitro approaches also provide a range for expected Hb saturation at a given P_O2_. Arterial Hb saturation profiles reconstructed with both approaches from P_O2_ profiles during sleep apnea should provide a valuable reference range with which to evaluate NIR estimates of arterial Hb saturation.

## Conclusions

Hemoximetry and blood gas analyses of blood samples from elephant seals during sleep apnea provided a valuable in vivo approach to construct Hill plot equations and ODCs, estimate P_50_s, document COHb and metHb contents, and calculate blood O_2_ depletion rates. The resulting ODC was shifted to the left and had a 3.4 mm lower P_50_ compared to those determined previously with an in vitro approach in the laboratory (mixing technique). Applying the in vivo and in vitro Hill plot equations to P_O2_ profiles during sleep apnea yielded similar estimates of blood O_2_ depletion rates because the in vivo approach had higher Hb saturations but lower functional Hb content. We conclude that, although COHb and metHb decrease the functional [Hb] available for O_2_ binding in elephant seals, the increased Hb-O_2_ affinity (lower P_50_) and Hb saturation calculated with the in vivo approach resulted in blood O_2_ contents at a given P_O2_ that were similar to those in comparison to that calculated with the in vitro approach differing by ≤ 1 ml O_2_ dl^− 1^ difference. Differences in mean blood O_2_ depletion rates during sleep apneas calculated with either approach were also minor, ≤ 0.4 ml O_2_ dl^− 1^ min^− 1^. Accordingly, application of either the in vitro or the in vivo Hill plot equations essentially yields the same results.

We also conclude that the higher and lower arterial Hb saturations calculated with the in vivo and in vitro approaches, respectively, are the most appropriate range of data to assess the validity and accuracy of non-invasive NIR-based measurements of arterial Hb saturations during sleep apnea.

We propose that sleep apnea in the elephant seal is an excellent model in which to investigate further the effect of COHb in O_2_ transport, blood O_2_ content and blood O_2_ depletion as well as the accuracy of newly developed NIR monitors of cerebral arterial Hb saturation. The differences between the in vitro and in vivo P_50_s and Hill plot equations may be secondary to methodological differences, including P_O2_ analyzer used, red blood cell DPG stability during time until analysis, and/or possible CO washout during tonometry in the mixing technique. This question could be addressed by analyzing fresh blood samples and tonometer-prepared samples from sleeping seals with the same hemoximeter / blood gas analyzer, a Tucker chamber for total blood O_2_ content, and a cyanomethemoglobin spectrophotometric analysis. Such an approach would allow documentation of blood gases, pH, COHb, metHb, total Hb, functional Hb, Hb saturation and total blood O_2_ content under directly comparable conditions, providing a definitive evaluation of methodological effects on estimates of O_2_ transport.

## Supplementary Information

Below is the link to the electronic supplementary material.

Supplementary Material 1

## Data Availability

Data are listed in Tables S2 and S3.

## Abbreviations

CO: Carbon monoxide
COHb: Carboxyhemoglobin
EDV: Extradural vein
Fe: Iron
Hb: Hemoglobin
HS: Hepatic sinus
IM: Intramuscular
kPa: kiloPascal
metHb: Methemoglobin
mm Hg: Millimeter of mercury
ODC: Oxygen-hemoglobin dissociation curve
P_50_: Partial pressure of oxygen at which hemoglobin is 50% saturated
P_CO2_: Partial pressure of carbon dioxide
P_O2_: Partial pressure of oxygen
S_O2_: Hemoglobin saturation
