# Supplementary material for: Carboxyhemoglobin and depletion of blood oxygen in sleeping elephant seals
Source: J Comp Physiol B. 2026 Jun 6;196(3):387–401. doi: 10.1007/s00360-026-01680-1 (PMC13282200; doi:10.1007/s00360-026-01680-1)
Supplement: Supplementary file 1 — Supplementary Material 1 [file 360_2026_1680_MOESM1_ESM.pdf]

Carboxyhemoglobin and depletion of blood oxygen in sleeping elephant seals

Journal of Comparative Physiology B

P. J. Ponganis[1*], B. I. McDonald[2], C. L. Williams[3], J. U. Meir[4], C. V. Brown[5], A. Patrician[5], J.C. Tremblay[6], A.G. Hindle[7], L.J. Pallin[8], J.M. Kendall-Bar[1], J.C. McKnight[9], D.P. Costa[7], T.M. Williams[7] and P.N. Ainslie[5].

[1]Center for Marine Biotechnology & Biomedicine, Scripps Institution of Oceanography, University of California San Diego, La Jolla, CA 92093-0204, USA, [2]Moss Landing Marine Laboratories, California State University, 8272 Moss Landing Road, Moss Landing, CA, 95039 USA, [3]National Marine Mammal Foundation, 2240 Shelter Island Drive, San Diego, CA 92106, USA, [4] NASA, Johnson Space Center, 2101 E. NASA Parkway, Houston, TX 77058, USA, [5]Centre for Heart, Lung and Vascular Health, School of Health and Exercise Sciences, University of British Columbia Okanagan, Kelowna, British Columbia, CA, [6]Cardiff School of Sport and Health Sciences, Cardiff Metropolitan University, Cardiff, Wales, UK, [7]School of Life Sciences, University of Nevada Las Vegas, 4505 Maryland Parkway, Las Vegas, NV 89154, USA, [8]Department of Ecology and Evolutionary Biology, University of California Santa Cruz, 130 McAllister Way, Santa Cruz, CA 95060, USA, [9]Sea Mammal Research Unit, Scottish Oceans Institute, University of St. Andrews, UK

*Author for correspondence: pponganis@ucsd.edu

Table S1. Analysis of paired arterial (art) and venous (ven) blood data and Hill equations and P_{50}s determined from the arterial and venous data. There were no striking differences between arterial and venous parameters (COHb, metHb, pH and P_{CO2}) that affect the oxygen affinity of hemoglobin (Hb) and the P_{50} (partial pressure of O_2 at 50% Hb saturation). The resulting P_{50}s in the individual seals and in all seals combined were near 27 mm Hg. Although differences were small, venous blood P_{50}s were higher (lower O_2 affinity) than that in arterial blood, consistent with the slightly lower pH in venous blood and slightly higher COHb level in arterial blood. Intercept (a) and slope (b) include (SE). Abbreviations: P_{CO2} – partial pressure of carbon dioxide, COHb – carboxyhemoglobin, metHb – methemoglobin.

	$COHb_{art}$	$COHb_{ven}$	$metHb_{art}$	$metHb_{ven}$	pH_{art}	pH_{ven}	P_{CO2art}	P_{CO2ven}
	**%**	**%**	**%**	**%**	**pH units**	**pH units**	**mm H**	**mm Hg**
**Seal 1**	**6.1**	**5.7**	**2.0**	**2.2**	**7.40**	**7.38**	**53**	**56**
**n = 14**	**0.05**	**0.06**	**0.20**	**0.02**	**0.004**	**0.002**	**0.9**	**0.5**
**Seal 3**	**4.8**	**4.7**	**2.2**	**2.2**	**7.37**	**7.36**	**56**	**56**
**n = 8**	**0.09**	**0.09**	**0.09**	**0.06**	**0.008**	**0.003**	**2.1**	**0.9**
**Seal 5**	**6.7**	**6.6**	**2.3**	**2.3**	**7.37**	**7.36**	**56**	**56**
**n = 5**	**0.14**	**0.13**	**0.07**	**0.03**	**0.009**	**0.004**	**2.8**	**3.1**
**In vivo arterial Hill equation & P_{50}**				**a**	**b**	**r^2**	**P_{50art}**	**n**
							**mm Hg**	
**Seal 1**				**2.87844 (0.28726)**	**-4.09800 (0.46898)**	**0.88**	**26.8**	**14**
**Seal 3**				**3.05217 (0.07576)**	**-4.37431 (0.10986)**	**0.99**	**27.1**	**8**
**Seal 5**				**3.48964 (0.1368)**	**-4.96724 (0.20958)**	**0.99**	**26.9**	**5**
**All seals**				**3.06382 (0.12169)**	**-4.38224 (0.19007)**	**0.96**	**27.0**	**27**
**In vivo venous Hill equation & P_{50}**				**a**	**b**	**r^2**	**P_{50ven}**	**n**
							**mm Hg**	
**Seal 1**				**2.87921 (0.06647)**	**-4.11936 (0.09779)**	**0.99**	**27.1**	**14**
**Seal 3**				**2.77300 (0.04346)**	**-4.00000 (0.06165)**	**0.99**	**27.9**	**8**
**Seal 5**				**2.96326 (0.07703)**	**-4.24025 (0.11438)**	**0.99**	**27.1**	**5**
**All seals**				**2.9128 (0.04902)**	**-4.17676 (0.07148)**	**0.99**	**27.5**	**27**

Fig. S1. Arterial, hepatic sinus (HS) and extradural vein (EDV) P_{O2} profiles with EDV pressure profiles during sleep apnea. Oscillations in EDV pressure profiles identify breaths. (A, C, E). Series of sleep apneas. (B, D, F). Single apnea.

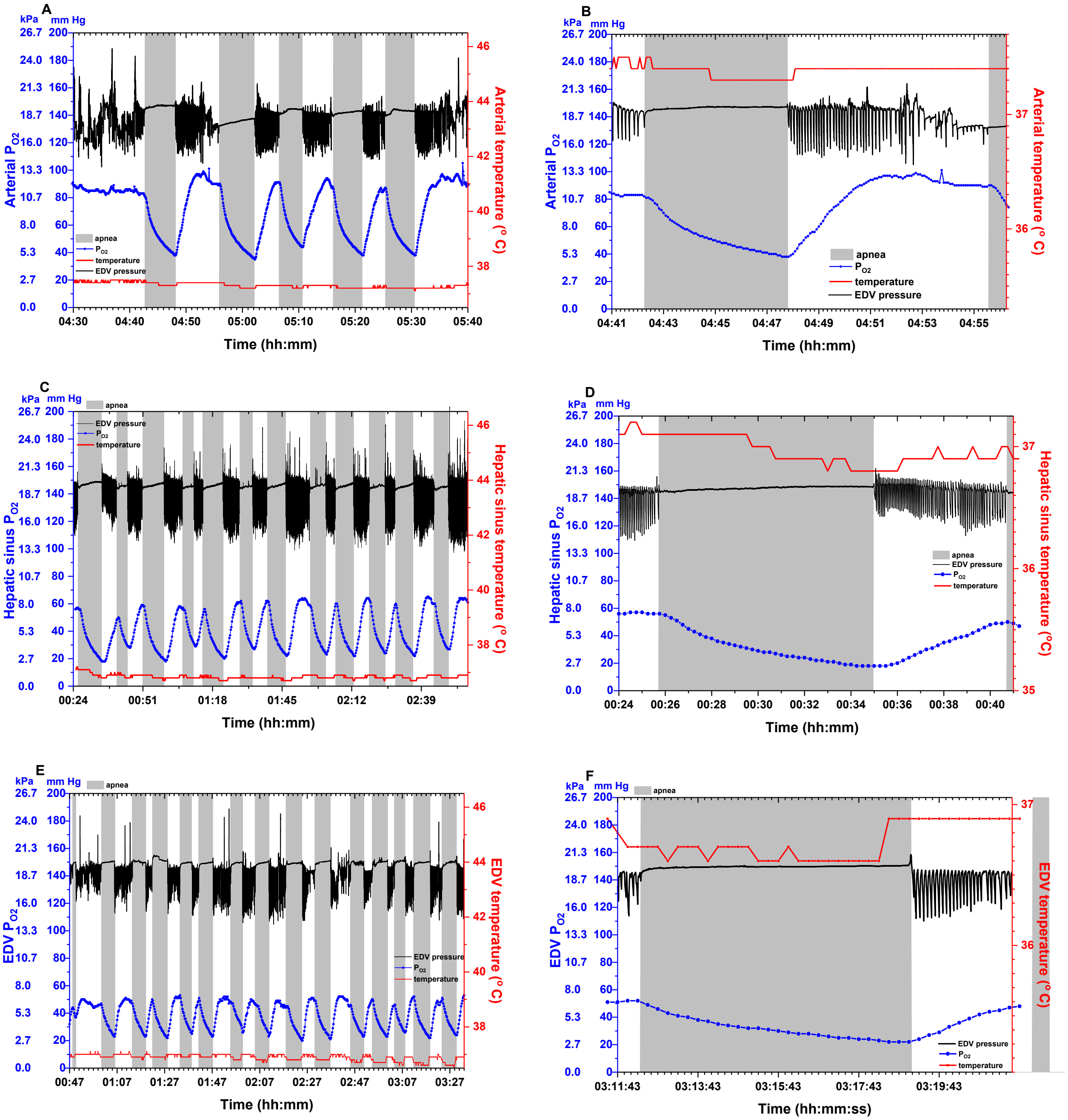

Table S2. Elephant seal blood data from the ABL90 Flex Plus hemoximeter in 2022.
Abbrfeviations: tHb - total hemoglobin, PO2 - partial pressure of oxygen, sO2 - Hb saturation, O2Hb - oxyHb, RHb - deoxyHb, COHb - carboxyhemoglobin, MetHb - methemoglobin, PCO2 - partial pressure of carbon dioxide, Lac - lactate concentration

seal #	sample_type		apnea vs eupnea	ABL90 date / time	tHb (g/dL)	PO2 (mmHg)	sO2 (%)	O2Hb (%)	RHb (%)	COHb (%)	MetHb (%)	pH	PCO2 (mmHg)	Lac (mmol/L)
1	Arterial	a01	ap	3/29/22 23:00	23.6	34.6	67.4	61.9	29.9	6.0	2.3	7.41	53.4	0.95
1	Arterial	a02	ap	3/29/22 23:53	23.6	34.8	68.1	62.5	29.3	6.0	2.3	7.40	54.8	1.05
1	Arterial	a03	ap	3/30/22 0:13	23.7	37.4	69.6	63.8	27.9	6.1	2.3	7.39	54.6	1.10
1	Arterial	a04	ap	3/30/22 0:19	23.4	39.7	72.7	66.8	25.1	6.0	2.1	7.38	58.0	1.17
1	Arterial	a05	ap	3/30/22 0:34	23.4	37.2	72.8	67.6	25.2	6.0	1.2	7.37	59.6	1.13
1	Arterial	a06	ap	3/30/22 1:27	23.1	36.0	71.5	65.5	26.1	6.1	2.4	7.41	51.4	1.05
1	Arterial	a07	ap	3/30/22 1:40	23.7	40.2	74.1	67.8	23.7	6.2	2.4	7.42	49.2	1.07
1	Arterial	a08	ap	3/30/22 2:06	26.2	68.4	89.5	81.4	9.6	6.6	2.4	7.40	52.0	1.20
1	Arterial	a09	ap	3/30/22 2:15	21.8	35.0	72.5	68.5	26.0	5.9	-0.3	7.40	53.1	1.15
1	Arterial	a10	ap	3/30/22 2:34	23.2	30.9	60.3	55.5	36.5	5.8	2.3	7.40	53.4	1.10
1	Venous	v01	ap	3/29/22 23:03	23.0	34.5	66.8	61.4	30.5	6.0	2.2	7.39	54.7	1.25
1	Venous	v02	ap	3/29/22 23:53	22.9	32.1	61.8	56.9	35.1	5.9	2.2	7.39	54.5	1.30
1	Venous	v03	ap	3/30/22 0:13	23.1	30.4	58.2	53.5	38.6	5.8	2.2	7.38	56.7	1.40
1	Venous	v04	ap	3/30/22 0:19	23.3	26.8	49.1	45.3	47.0	5.6	2.2	7.37	58.4	1.40
1	Venous	v05	ap	3/30/22 0:34	22.9	26.5	48.0	44.4	48.1	5.5	2.1	7.37	57.2	1.35
1	Venous	v06	ap	3/30/22 1:27	23.0	26.1	49.1	45.4	47.0	5.5	2.2	7.40	54.7	1.30
1	Venous	v07	ap	3/30/22 1:40	23.2	27.6	51.6	47.5	44.6	5.7	2.2	7.38	57.3	1.20
1	Venous	v08	ap	3/30/22 2:06	23.7	26.6	48.9	45.2	47.2	5.6	2.1	7.38	58.5	1.20
1	Venous	v09	ap	3/30/22 2:15	23.3	26.5	48.6	44.8	47.4	5.6	2.2	7.38	58.2	1.20
1	Venous	v10	ap	3/30/22 2:37	22.8	23.6	40.9	37.8	54.8	5.3	2.2	7.37	59.4	1.20
1	Arterial	a01	eup	3/29/22 20:18	24.8	54.2	89.8	82.2	9.4	6.1	2.4	7.41	49.3	1.60
1	Arterial	a02	eup	3/29/22 21:24	23.8	52.9	89.4	82.0	9.8	6.0	2.3	7.40	50.9	1.40
1	Arterial	a03	eup	3/29/22 23:42	23.6	54.6	90.1	82.6	9.1	6.0	2.4	7.41	49.0	1.10
1	Arterial	a04	eup	3/30/22 0:01	22.7	58.6	94.3	86.7	5.2	6.3	1.9	7.42	48.1	1.05
1	Venous	v01	eup	3/29/22 20:18	23.3	39.9	76.8	70.3	21.2	6.2	2.4	7.40	52.6	1.75
1	Venous	v02	eup	3/29/22 21:24	23.5	35.4	68.3	62.6	29.2	6.0	2.3	7.39	54.6	1.50
1	Venous	v03	eup	3/29/22 23:42	23.1	33.3	63.4	58.2	33.7	5.9	2.2	7.38	57.7	1.20
1	Venous	v04	eup	3/30/22 0:01	22.6	28.5	54.3	50.1	42.2	5.6	2.2	7.38	55.8	1.30

seal #	sample_type		apnea vs eupnea	ABL90 time	tHb (g/dL)	pO2 (mmHg)	sO2 (%)	O2Hb (%)	RHb (%)	COHb (%)	MetHb (%)	pH	pCO2 (mmHg)	Lac (mmol/L)
3	Arterial	a01	ap	4/2/22 20:49	22.1	27.4	48.3	45.0	48.1	4.8	2.2	7.37	61.7	0.80
3	Arterial	a02	ap	4/2/22 21:07	21.9	29.0	53.4	49.6	43.3	4.9	2.3	7.39	56.2	0.80
3	Arterial	a03	ap	4/2/22 22:16	22.3	24.2	41.3	38.5	54.6	4.7	2.2	7.38	59.7	0.90
3	Arterial	a04	ap	4/2/22 22:46	22.6	22.9	37.1	34.6	58.7	4.6	2.2	7.37	62.4	0.95
3	Arterial	a04.9	ap	4/2/22 22:49	23.1	21.2	33.1	30.9	62.5	4.5	2.2	7.37	63.3	1.00
3	Arterial	a05.8	ap	4/2/22 23:50	21.9	26.8	49.5	46.0	46.9	4.9	2.3	7.38	59.6	0.90
3	Arterial	a05.9	ap	4/2/22 23:57	20.8	25.6	47.5	44.4	49.1	4.8	1.8	7.36	64.0	1.00
3	Venous	v01	ap	4/2/22 20:49	21.8	24.8	41.3	38.5	54.8	4.6	2.2	7.36	61.8	1.15
3	Venous	v02	ap	4/2/22 21:07	21.3	28.1	50.8	47.2	45.7	4.8	2.3	7.37	59.1	1.10
3	Venous	v03	ap	4/2/22 22:16	22.3	24.1	40.7	37.9	55.4	4.7	2.2	7.36	61.8	1.10
3	Venous	v04	ap	4/2/22 22:46	22.5	23.1	37.9	35.4	58.0	4.6	2.1	7.36	61.9	1.20
3	Venous	v04.9	ap	4/2/22 22:49	22.8	21.1	32.7	30.6	62.9	4.5	2.1	7.36	63.5	1.30
3	Venous	v05.8	ap	4/2/22 23:50	22.1	25.2	42.7	39.8	53.4	4.7	2.1	7.36	62.8	1.20
3	Venous	v05.9	ap	4/2/22 23:57	23.7	23.0	37.6	35.0	58.3	4.7	2.1	7.35	63.4	1.20
3	Arterial		eup	4/2/22 15:54	21.7	56.9	90.8	83.5	8.5	5.3	2.7	7.37	54.3	1.60
3	Venous		eup	4/2/22 15:54	22.2	44.7	79.2	73.0	19.2	5.3	2.6	7.36	56.8	1.75

seal #	sample_type		apnea vs eupnea	ABL90 time	tHb (g/dL)	pO2 (mmHg)	sO2 (%)	O2Hb (%)	RHb (%)	COHb (%)	MetHb (%)	pH	pCO2 (mmHg)	Lac (mmol/L)
5	Arterial	a01	ap	4/8/22 21:06	21.2	30.8	60.4	55.1	36.1	6.7	2.2	7.36	55.8	0.80
5	Arterial	a02	ap	4/8/22 21:13	22.8	30.4	61.1	55.6	35.4	6.8	2.4	7.37	57.5	0.80
5	Arterial	ao3	ap	4/8/22 22:03	22.0	24.7	47.2	43.1	48.3	6.4	2.2	7.36	57.8	0.85
5	Arterial	ao4	ap	4/8/22 22:18	21.7	27.4	51.6	47.0	44.2	6.5	2.3	7.36	58.2	0.80
5	Venous	v01	ap	4/8/22 21:06	22.2	28.7	53.7	48.9	42.3	6.6	2.3	7.35	56.2	1.15
5	Venous	v02	ap	4/8/22 21:16	22.3	29.7	56.3	51.3	39.8	6.6	2.3	7.36	55.9	1.10
5	Venous	v03	ap	4/8/22 22:06	21.7	24.6	44.2	40.4	51.0	6.4	2.3	7.35	57.7	1.20
5	Venous	v04	ap	4/8/22 22:18	21.6	27.7	52.1	47.5	43.8	6.5	2.3	7.35	57.9	1.10
5	Arterial		eup	4/8/22 20:59	22.1	66.3	96.2	86.8	3.4	7.2	2.6	7.40	46.8	0.85
5	Venous		eup	4/8/22 20:59	22.0	44.6	81.9	74.1	16.4	7.1	2.5	7.37	53.1	1.25

Table S3. Sleep apnea PO2 and temperature profile data (date/time, PO2) from elephant seals collected in 2006-2008 from Meir et al. 2009. See text for details.
Data are from the aorta, hepatic sinus and extradural vein in three seals. Apneic intervals are listed below each data set.
PO2 is in mm Hg., temperature in degrees C. Abbreviation: PO2 - partial pressure of oxygen.

Arterial Profile			Hepatlic Sinus Profile			Extradural Vein Profile		
Date / time	PO2	Temperature	Date / time	PO2	Temperature	Date / time	PO2	Temperature
4/14/2008 4:30:00	89	37.5	4/19/2007 0:24:49	56	37.1	4/23/2006 0:47:13	32	37
4/14/2008 4:30:05	89	37.5	4/19/2007 0:25:04	56	37.1	4/23/2006 0:47:28	35	37
4/14/2008 4:30:10	89	37.4	4/19/2007 0:25:19	57	37.2	4/23/2006 0:47:43	39	36.9
4/14/2008 4:30:15	89	37.5	4/19/2007 0:25:34	57	37.2	4/23/2006 0:47:58	41	37
4/14/2008 4:30:20	88	37.4	4/19/2007 0:25:49	57	37.1	4/23/2006 0:48:13	43	36.9
4/14/2008 4:30:25	88	37.5	4/19/2007 0:26:04	56	37.1	4/23/2006 0:48:28	44	36.9
4/14/2008 4:30:30	88	37.4	4/19/2007 0:26:19	56	37.1	4/23/2006 0:48:43	44	36.9
4/14/2008 4:30:35	87	37.5	4/19/2007 0:26:34	56	37.1	4/23/2006 0:48:58	43	36.9
4/14/2008 4:30:40	87	37.4	4/19/2007 0:26:49	55	37.1	4/23/2006 0:49:13	40	36.9
4/14/2008 4:30:45	87	37.5	4/19/2007 0:27:04	53	37.1	4/23/2006 0:49:28	38	37
4/14/2008 4:30:50	87	37.4	4/19/2007 0:27:19	51	37.1	4/23/2006 0:49:43	37	37
4/14/2008 4:30:55	87	37.4	4/19/2007 0:27:34	48	37.1	4/23/2006 0:49:58	37	37
4/14/2008 4:31:00	88	37.4	4/19/2007 0:27:49	45	37.1	4/23/2006 0:50:13	39	37
4/14/2008 4:31:05	88	37.5	4/19/2007 0:28:04	43	37.1	4/23/2006 0:50:28	40	37
4/14/2008 4:31:10	89	37.5	4/19/2007 0:28:19	41	37.1	4/23/2006 0:50:43	42	37
4/14/2008 4:31:15	89	37.5	4/19/2007 0:28:34	39	37.1	4/23/2006 0:50:58	44	37
4/14/2008 4:31:20	89	37.5	4/19/2007 0:28:49	38	37.1	4/23/2006 0:51:13	46	37
4/14/2008 4:31:25	89	37.5	4/19/2007 0:29:04	36	37.1	4/23/2006 0:51:28	47	37
4/14/2008 4:31:30	88	37.4	4/19/2007 0:29:19	35	37.1	4/23/2006 0:51:43	48	37
4/14/2008 4:31:35	87	37.5	4/19/2007 0:29:34	34	37.1	4/23/2006 0:51:58	49	37
4/14/2008 4:31:40	87	37.5	4/19/2007 0:29:49	33	37.1	4/23/2006 0:52:13	50	37
4/14/2008 4:31:45	87	37.4	4/19/2007 0:30:04	32	37.1	4/23/2006 0:52:28	50	37
4/14/2008 4:31:50	86	37.5	4/19/2007 0:30:19	31	37.1	4/23/2006 0:52:43	50	37
4/14/2008 4:31:55	86	37.4	4/19/2007 0:30:34	30	37	4/23/2006 0:52:58	50	37
4/14/2008 4:32:00	86	37.5	4/19/2007 0:30:49	29	37	4/23/2006 0:53:13	50	37
4/14/2008 4:32:05	86	37.5	4/19/2007 0:31:04	28	37	4/23/2006 0:53:28	50	37
4/14/2008 4:32:10	86	37.4	4/19/2007 0:31:19	28	37	4/23/2006 0:53:43	49	37
4/14/2008 4:32:15	85	37.4	4/19/2007 0:31:34	27	36.9	4/23/2006 0:53:58	49	37
4/14/2008 4:32:20	85	37.4	4/19/2007 0:31:49	26	36.9	4/23/2006 0:54:13	48	37
4/14/2008 4:32:25	85	37.4	4/19/2007 0:32:04	25	36.9	4/23/2006 0:54:28	48	37
4/14/2008 4:32:30	85	37.5	4/19/2007 0:32:19	25	36.9	4/23/2006 0:54:43	48	37
4/14/2008 4:32:35	85	37.4	4/19/2007 0:32:34	24	36.9	4/23/2006 0:54:58	47	37
4/14/2008 4:32:40	85	37.5	4/19/2007 0:32:49	24	36.9	4/23/2006 0:55:13	47	37
4/14/2008 4:32:45	84	37.5	4/19/2007 0:33:04	23	36.9	4/23/2006 0:55:28	47	37
4/14/2008 4:32:50	85	37.4	4/19/2007 0:33:19	22	36.9	4/23/2006 0:55:43	48	37
4/14/2008 4:32:55	85	37.4	4/19/2007 0:33:34	22	36.9	4/23/2006 0:55:58	47	37.1
4/14/2008 4:33:00	85	37.4	4/19/2007 0:33:49	21	36.8	4/23/2006 0:56:13	47	37
4/14/2008 4:33:05	85	37.4	4/19/2007 0:34:04	21	36.9	4/23/2006 0:56:28	46	37
4/14/2008 4:33:10	85	37.5	4/19/2007 0:34:19	20	36.9	4/23/2006 0:56:43	46	37
4/14/2008 4:33:15	85	37.4	4/19/2007 0:34:34	19	36.9	4/23/2006 0:56:58	45	37
4/14/2008 4:33:20	85	37.4	4/19/2007 0:34:49	19	36.8	4/23/2006 0:57:13	44	37
4/14/2008 4:33:25	85	37.4	4/19/2007 0:35:04	18	36.8	4/23/2006 0:57:28	44	37
4/14/2008 4:33:30	85	37.4	4/19/2007 0:35:19	18	36.8	4/23/2006 0:57:43	44	37
4/14/2008 4:33:35	85	37.4	4/19/2007 0:35:34	18	36.8	4/23/2006 0:57:58	45	37.1
4/14/2008 4:33:40	85	37.4	4/19/2007 0:35:49	18	36.8	4/23/2006 0:58:13	45	37
4/14/2008 4:33:45	85	37.4	4/19/2007 0:36:04	18	36.8	4/23/2006 0:58:28	45	37
4/14/2008 4:33:50	85	37.4	4/19/2007 0:36:19	18	36.8	4/23/2006 0:58:43	45	37
4/14/2008 4:33:55	85	37.5	4/19/2007 0:36:34	19	36.8	4/23/2006 0:58:58	45	37
4/14/2008 4:34:00	85	37.4	4/19/2007 0:36:49	20	36.8	4/23/2006 0:59:13	46	37.1
4/14/2008 4:34:05	86	37.4	4/19/2007 0:37:04	22	36.9	4/23/2006 0:59:28	46	37
4/14/2008 4:34:10	86	37.5	4/19/2007 0:37:19	24	36.9	4/23/2006 0:59:43	46	37
4/14/2008 4:34:15	86	37.4	4/19/2007 0:37:34	26	36.9	4/23/2006 0:59:58	46	37
4/14/2008 4:34:20	86	37.4	4/19/2007 0:37:49	28	36.9	4/23/2006 1:00:13	46	36.9
4/14/2008 4:34:25	85	37.4	4/19/2007 0:38:04	30	36.9	4/23/2006 1:00:28	47	36.9
4/14/2008 4:34:30	85	37.4	4/19/2007 0:38:19	31	36.9	4/23/2006 1:00:43	46	36.9
4/14/2008 4:34:35	85	37.4	4/19/2007 0:38:34	33	37	4/23/2006 1:00:58	44	36.9
4/14/2008 4:34:40	84	37.4	4/19/2007 0:38:49	35	36.9	4/23/2006 1:01:13	42	36.9
4/14/2008 4:34:45	84	37.4	4/19/2007 0:39:04	36	36.9	4/23/2006 1:01:28	40	36.9
4/14/2008 4:34:50	84	37.4	4/19/2007 0:39:19	38	36.9	4/23/2006 1:01:43	38	36.9
4/14/2008 4:34:55	84	37.4	4/19/2007 0:39:34	39	36.9	4/23/2006 1:01:58	37	36.9
4/14/2008 4:35:00	84	37.5	4/19/2007 0:39:49	40	36.9	4/23/2006 1:02:13	36	36.9
4/14/2008 4:35:05	85	37.5	4/19/2007 0:40:04	42	37	4/23/2006 1:02:28	34	36.9
4/14/2008 4:35:10	85	37.5	4/19/2007 0:40:19	44	36.9	4/23/2006 1:02:43	34	36.9
4/14/2008 4:35:15	85	37.4	4/19/2007 0:40:34	46	36.9	4/23/2006 1:02:58	32	36.9
4/14/2008 4:35:20	85	37.4	4/19/2007 0:40:49	48	36.9	4/23/2006 1:03:13	31	36.9
4/14/2008 4:35:25	85	37.5	4/19/2007 0:41:04	49	36.9	4/23/2006 1:03:28	31	36.9
4/14/2008 4:35:30	84	37.4	4/19/2007 0:41:19	49	37	4/23/2006 1:03:43	30	36.9
4/14/2008 4:35:35	84	37.4	4/19/2007 0:41:34	50	37	4/23/2006 1:03:58	28	36.9
4/14/2008 4:35:40	83	37.4	4/19/2007 0:41:49	49	36.9	4/23/2006 1:04:13	28	36.9
4/14/2008 4:35:45	82	37.5	4/19/2007 0:42:04	47	36.9	4/23/2006 1:04:28	27	36.9
4/14/2008 4:35:50	83	37.4	4/19/2007 0:42:19	44	37	4/23/2006 1:04:43	27	36.9
4/14/2008 4:35:55	83	37.4	4/19/2007 0:42:34	42	36.9	4/23/2006 1:04:58	26	36.9
4/14/2008 4:36:00	83	37.4	4/19/2007 0:42:49	40	36.9	4/23/2006 1:05:13	25	36.9
4/14/2008 4:36:05	83	37.4	4/19/2007 0:43:04	38	36.9	4/23/2006 1:05:28	24	36.9
4/14/2008 4:36:10	83	37.5	4/19/2007 0:43:19	37	36.9	4/23/2006 1:05:43	24	37
4/14/2008 4:36:15	83	37.4	4/19/2007 0:43:34	36	36.9	4/23/2006 1:05:58	23	37
4/14/2008 4:36:20	84	37.4	4/19/2007 0:43:49	34	36.9	4/23/2006 1:06:13	23	37
4/14/2008 4:36:25	84	37.4	4/19/2007 0:44:04	33	36.8	4/23/2006 1:06:28	25	37
4/14/2008 4:36:30	85	37.4	4/19/2007 0:44:19	32	36.8	4/23/2006 1:06:43	28	37
4/14/2008 4:36:35	86	37.5	4/19/2007 0:44:34	31	36.8	4/23/2006 1:06:58	31	37

4/14/2008 4:36:40	87	37.5	4/19/2007 0:44:49	30	36.8	4/23/2006 1:07:13	35	37
4/14/2008 4:36:45	88	37.5	4/19/2007 0:45:04	29	36.8	4/23/2006 1:07:28	38	37
4/14/2008 4:36:50	88	37.5	4/19/2007 0:45:19	29	36.8	4/23/2006 1:07:43	41	37
4/14/2008 4:36:55	87	37.5	4/19/2007 0:45:34	29	36.8	4/23/2006 1:07:58	42	37
4/14/2008 4:37:00	86	37.5	4/19/2007 0:45:49	28	36.8	4/23/2006 1:08:13	45	37
4/14/2008 4:37:05	86	37.5	4/19/2007 0:46:04	28	36.8	4/23/2006 1:08:28	45	37
4/14/2008 4:37:10	89	37.5	4/19/2007 0:46:19	29	36.9	4/23/2006 1:08:43	46	37.1
4/14/2008 4:37:15	85	37.5	4/19/2007 0:46:34	31	36.9	4/23/2006 1:08:58	47	37
4/14/2008 4:37:20	85	37.5	4/19/2007 0:46:49	32	36.8	4/23/2006 1:09:13	47	37
4/14/2008 4:37:25	85	37.5	4/19/2007 0:47:04	34	36.9	4/23/2006 1:09:28	48	37
4/14/2008 4:37:30	85	37.5	4/19/2007 0:47:19	36	36.9	4/23/2006 1:09:43	49	37
4/14/2008 4:37:35	84	37.5	4/19/2007 0:47:34	39	36.9	4/23/2006 1:09:58	49	37
4/14/2008 4:37:40	85	37.5	4/19/2007 0:47:49	41	36.9	4/23/2006 1:10:13	50	37
4/14/2008 4:37:45	85	37.5	4/19/2007 0:48:04	44	36.9	4/23/2006 1:10:28	50	37
4/14/2008 4:37:50	85	37.5	4/19/2007 0:48:19	46	36.9	4/23/2006 1:10:43	51	37
4/14/2008 4:37:55	85	37.5	4/19/2007 0:48:34	47	36.9	4/23/2006 1:10:58	51	37
4/14/2008 4:38:00	85	37.5	4/19/2007 0:48:49	49	36.9	4/23/2006 1:11:13	50	37
4/14/2008 4:38:05	85	37.5	4/19/2007 0:49:04	52	36.9	4/23/2006 1:11:28	50	37
4/14/2008 4:38:10	85	37.4	4/19/2007 0:49:19	52	36.9	4/23/2006 1:11:43	50	37
4/14/2008 4:38:15	84	37.5	4/19/2007 0:49:34	54	36.9	4/23/2006 1:11:58	50	37
4/14/2008 4:38:20	85	37.5	4/19/2007 0:49:49	56	36.9	4/23/2006 1:12:13	50	37.1
4/14/2008 4:38:25	85	37.5	4/19/2007 0:50:04	57	36.9	4/23/2006 1:12:28	49	37.1
4/14/2008 4:38:30	84	37.5	4/19/2007 0:50:19	58	36.9	4/23/2006 1:12:43	48	37
4/14/2008 4:38:35	84	37.5	4/19/2007 0:50:34	59	36.9	4/23/2006 1:12:58	49	37
4/14/2008 4:38:40	84	37.5	4/19/2007 0:50:49	59	36.9	4/23/2006 1:13:13	49	36.9
4/14/2008 4:38:45	84	37.5	4/19/2007 0:51:04	59	36.9	4/23/2006 1:13:28	47	36.9
4/14/2008 4:38:50	84	37.5	4/19/2007 0:51:19	58	36.9	4/23/2006 1:13:43	47	36.9
4/14/2008 4:38:55	84	37.5	4/19/2007 0:51:34	58	36.9	4/23/2006 1:13:58	45	36.9
4/14/2008 4:39:00	84	37.4	4/19/2007 0:51:49	55	36.9	4/23/2006 1:14:13	43	36.9
4/14/2008 4:39:05	85	37.4	4/19/2007 0:52:04	52	36.9	4/23/2006 1:14:28	41	36.9
4/14/2008 4:39:10	85	37.5	4/19/2007 0:52:19	49	36.9	4/23/2006 1:14:43	40	36.9
4/14/2008 4:39:15	85	37.5	4/19/2007 0:52:34	46	36.9	4/23/2006 1:14:58	38	36.9
4/14/2008 4:39:20	86	37.5	4/19/2007 0:52:49	44	36.9	4/23/2006 1:15:13	36	36.9
4/14/2008 4:39:25	86	37.4	4/19/2007 0:53:04	42	36.9	4/23/2006 1:15:28	35	36.9
4/14/2008 4:39:30	86	37.5	4/19/2007 0:53:19	40	36.9	4/23/2006 1:15:43	34	36.9
4/14/2008 4:39:35	86	37.4	4/19/2007 0:53:34	38	36.9	4/23/2006 1:15:58	33	36.9
4/14/2008 4:39:40	85	37.5	4/19/2007 0:53:49	36	36.9	4/23/2006 1:16:13	33	36.9
4/14/2008 4:39:45	86	37.5	4/19/2007 0:54:04	35	36.9	4/23/2006 1:16:28	31	36.9
4/14/2008 4:39:50	86	37.5	4/19/2007 0:54:19	33	36.9	4/23/2006 1:16:43	30	36.9
4/14/2008 4:39:55	86	37.4	4/19/2007 0:54:34	32	36.9	4/23/2006 1:16:58	30	36.9
4/14/2008 4:40:00	86	37.4	4/19/2007 0:54:49	32	36.8	4/23/2006 1:17:13	29	36.9
4/14/2008 4:40:05	86	37.4	4/19/2007 0:55:04	30	36.8	4/23/2006 1:17:28	28	36.9
4/14/2008 4:40:10	85	37.5	4/19/2007 0:55:19	29	36.8	4/23/2006 1:17:43	27	36.8
4/14/2008 4:40:15	85	37.4	4/19/2007 0:55:34	28	36.8	4/23/2006 1:17:58	26	36.9
4/14/2008 4:40:20	85	37.5	4/19/2007 0:55:49	27	36.8	4/23/2006 1:18:13	25	36.8
4/14/2008 4:40:25	85	37.4	4/19/2007 0:56:04	26	36.8	4/23/2006 1:18:28	24	36.9
4/14/2008 4:40:30	85	37.5	4/19/2007 0:56:19	26	36.8	4/23/2006 1:18:43	24	36.9
4/14/2008 4:40:35	85	37.4	4/19/2007 0:56:34	25	36.8	4/23/2006 1:18:58	23	37
4/14/2008 4:40:40	84	37.5	4/19/2007 0:56:49	24	36.8	4/23/2006 1:19:13	23	37
4/14/2008 4:40:45	84	37.4	4/19/2007 0:57:04	24	36.8	4/23/2006 1:19:28	24	37
4/14/2008 4:40:50	88	37.5	4/19/2007 0:57:19	23	36.8	4/23/2006 1:19:43	27	37.1
4/14/2008 4:40:55	85	37.5	4/19/2007 0:57:34	23	36.8	4/23/2006 1:19:58	30	37
4/14/2008 4:41:00	85	37.5	4/19/2007 0:57:49	22	36.8	4/23/2006 1:20:13	33	37
4/14/2008 4:41:05	86	37.5	4/19/2007 0:58:04	21	36.8	4/23/2006 1:20:28	36	37
4/14/2008 4:41:10	86	37.5	4/19/2007 0:58:19	21	36.8	4/23/2006 1:20:43	39	37
4/14/2008 4:41:15	85	37.4	4/19/2007 0:58:34	21	36.8	4/23/2006 1:20:58	42	37
4/14/2008 4:41:20	84	37.4	4/19/2007 0:58:49	20	36.7	4/23/2006 1:21:13	44	36.9
4/14/2008 4:41:25	84	37.5	4/19/2007 0:59:04	20	36.8	4/23/2006 1:21:28	46	36.9
4/14/2008 4:41:30	83	37.4	4/19/2007 0:59:19	19	36.7	4/23/2006 1:21:43	48	36.9
4/14/2008 4:41:35	83	37.5	4/19/2007 0:59:34	19	36.7	4/23/2006 1:21:58	50	36.9
4/14/2008 4:41:40	82	37.5	4/19/2007 0:59:49	18	36.8	4/23/2006 1:22:13	49	36.9
4/14/2008 4:41:45	82	37.5	4/19/2007 1:00:04	19	36.8	4/23/2006 1:22:28	47	36.9
4/14/2008 4:41:50	83	37.5	4/19/2007 1:00:19	19	36.8	4/23/2006 1:22:43	45	36.9
4/14/2008 4:41:55	83	37.5	4/19/2007 1:00:34	21	36.8	4/23/2006 1:22:58	43	36.9
4/14/2008 4:42:00	83	37.5	4/19/2007 1:00:49	22	36.8	4/23/2006 1:23:13	41	36.9
4/14/2008 4:42:05	83	37.4	4/19/2007 1:01:04	25	36.8	4/23/2006 1:23:28	39	36.9
4/14/2008 4:42:10	83	37.4	4/19/2007 1:01:19	26	36.8	4/23/2006 1:23:43	37	36.9
4/14/2008 4:42:15	83	37.4	4/19/2007 1:01:34	29	36.8	4/23/2006 1:23:58	36	36.9
4/14/2008 4:42:20	83	37.4	4/19/2007 1:01:49	31	36.8	4/23/2006 1:24:13	35	36.9
4/14/2008 4:42:25	83	37.5	4/19/2007 1:02:04	33	36.8	4/23/2006 1:24:28	34	36.9
4/14/2008 4:42:30	83	37.4	4/19/2007 1:02:19	36	36.8	4/23/2006 1:24:43	33	36.9
4/14/2008 4:42:35	82	37.4	4/19/2007 1:02:34	40	36.8	4/23/2006 1:24:58	32	36.9
4/14/2008 4:42:40	81	37.5	4/19/2007 1:02:49	42	36.8	4/23/2006 1:25:13	30	36.9
4/14/2008 4:42:45	81	37.5	4/19/2007 1:03:04	45	36.8	4/23/2006 1:25:28	30	36.9
4/14/2008 4:42:50	80	37.5	4/19/2007 1:03:19	48	36.8	4/23/2006 1:25:43	29	36.9
4/14/2008 4:42:55	79	37.4	4/19/2007 1:03:34	49	36.8	4/23/2006 1:25:58	28	36.9
4/14/2008 4:43:00	77	37.4	4/19/2007 1:03:49	52	36.8	4/23/2006 1:26:13	27	36.9
4/14/2008 4:43:05	76	37.4	4/19/2007 1:04:04	53	36.8	4/23/2006 1:26:28	27	36.9
4/14/2008 4:43:10	73	37.4	4/19/2007 1:04:19	55	36.8	4/23/2006 1:26:43	26	36.9
4/14/2008 4:43:15	72	37.4	4/19/2007 1:04:34	56	36.8	4/23/2006 1:26:58	25	36.9
4/14/2008 4:43:20	70	37.4	4/19/2007 1:04:49	57	36.8	4/23/2006 1:27:13	24	36.9
4/14/2008 4:43:25	68	37.4	4/19/2007 1:05:04	58	36.8	4/23/2006 1:27:28	24	36.8
4/14/2008 4:43:30	67	37.4	4/19/2007 1:05:19	58	36.9	4/23/2006 1:27:43	23	36.9
4/14/2008 4:43:35	65	37.4	4/19/2007 1:05:34	58	36.9	4/23/2006 1:27:58	23	37
4/14/2008 4:43:40	64	37.4	4/19/2007 1:05:49	57	36.9	4/23/2006 1:28:13	22	37
4/14/2008 4:43:45	63	37.4	4/19/2007 1:06:04	57	36.9	4/23/2006 1:28:28	22	36.9
4/14/2008 4:43:50	62	37.4	4/19/2007 1:06:19	56	36.9	4/23/2006 1:28:43	24	37

4/14/2008 4:43:55	61	37.4	4/19/2007 1:06:34	57	36.9	4/23/2006 1:28:58	27	37
4/14/2008 4:44:00	60	37.4	4/19/2007 1:06:49	56	36.8	4/23/2006 1:29:13	29	37
4/14/2008 4:44:05	59	37.4	4/19/2007 1:07:04	55	36.9	4/23/2006 1:29:28	32	37
4/14/2008 4:44:10	58	37.4	4/19/2007 1:07:19	55	36.9	4/23/2006 1:29:43	35	37
4/14/2008 4:44:15	58	37.4	4/19/2007 1:07:34	53	36.9	4/23/2006 1:29:58	39	37
4/14/2008 4:44:20	57	37.4	4/19/2007 1:07:49	50	36.9	4/23/2006 1:30:13	41	37
4/14/2008 4:44:25	56	37.4	4/19/2007 1:08:04	47	36.8	4/23/2006 1:30:28	45	37
4/14/2008 4:44:30	55	37.4	4/19/2007 1:08:19	45	36.8	4/23/2006 1:30:43	47	37
4/14/2008 4:44:35	55	37.4	4/19/2007 1:08:34	43	36.8	4/23/2006 1:30:58	48	37
4/14/2008 4:44:40	54	37.4	4/19/2007 1:08:49	41	36.8	4/23/2006 1:31:13	49	37
4/14/2008 4:44:45	53	37.4	4/19/2007 1:09:04	39	36.8	4/23/2006 1:31:28	51	36.9
4/14/2008 4:44:50	53	37.4	4/19/2007 1:09:19	37	36.8	4/23/2006 1:31:43	51	37
4/14/2008 4:44:55	52	37.4	4/19/2007 1:09:34	36	36.8	4/23/2006 1:31:58	52	37
4/14/2008 4:45:00	52	37.4	4/19/2007 1:09:49	35	36.8	4/23/2006 1:32:13	51	37
4/14/2008 4:45:05	51	37.4	4/19/2007 1:10:04	34	36.8	4/23/2006 1:32:28	51	36.9
4/14/2008 4:45:10	51	37.3	4/19/2007 1:10:19	33	36.8	4/23/2006 1:32:43	51	37
4/14/2008 4:45:15	50	37.3	4/19/2007 1:10:34	32	36.8	4/23/2006 1:32:58	52	37
4/14/2008 4:45:20	50	37.3	4/19/2007 1:10:49	31	36.8	4/23/2006 1:33:13	51	37
4/14/2008 4:45:25	49	37.3	4/19/2007 1:11:04	30	36.8	4/23/2006 1:33:28	52	37
4/14/2008 4:45:30	49	37.3	4/19/2007 1:11:19	29	36.8	4/23/2006 1:33:43	53	37
4/14/2008 4:45:35	48	37.3	4/19/2007 1:11:34	29	36.8	4/23/2006 1:33:58	51	36.9
4/14/2008 4:45:40	48	37.3	4/19/2007 1:11:49	30	36.8	4/23/2006 1:34:13	48	37
4/14/2008 4:45:45	48	37.3	4/19/2007 1:12:04	31	36.8	4/23/2006 1:34:28	46	36.9
4/14/2008 4:45:50	47	37.3	4/19/2007 1:12:19	33	36.8	4/23/2006 1:34:43	43	36.9
4/14/2008 4:45:55	47	37.3	4/19/2007 1:12:34	34	36.8	4/23/2006 1:34:58	41	36.9
4/14/2008 4:46:00	46	37.3	4/19/2007 1:12:49	36	36.8	4/23/2006 1:35:13	40	36.9
4/14/2008 4:46:05	46	37.3	4/19/2007 1:13:04	39	36.8	4/23/2006 1:35:28	38	36.9
4/14/2008 4:46:10	45	37.3	4/19/2007 1:13:19	42	36.9	4/23/2006 1:35:43	36	36.9
4/14/2008 4:46:15	45	37.3	4/19/2007 1:13:34	45	36.9	4/23/2006 1:35:58	36	36.9
4/14/2008 4:46:20	45	37.3	4/19/2007 1:13:49	47	36.8	4/23/2006 1:36:13	34	36.9
4/14/2008 4:46:25	44	37.3	4/19/2007 1:14:04	50	36.8	4/23/2006 1:36:28	33	36.9
4/14/2008 4:46:30	44	37.3	4/19/2007 1:14:19	52	36.9	4/23/2006 1:36:43	32	36.9
4/14/2008 4:46:35	44	37.3	4/19/2007 1:14:34	54	36.8	4/23/2006 1:36:58	31	36.9
4/14/2008 4:46:40	43	37.3	4/19/2007 1:14:49	56	36.8	4/23/2006 1:37:13	30	36.9
4/14/2008 4:46:45	43	37.3	4/19/2007 1:15:04	56	36.9	4/23/2006 1:37:28	29	37
4/14/2008 4:46:50	43	37.3	4/19/2007 1:15:19	54	36.9	4/23/2006 1:37:43	28	36.9
4/14/2008 4:46:55	42	37.3	4/19/2007 1:15:34	52	36.8	4/23/2006 1:37:58	28	36.9
4/14/2008 4:47:00	42	37.3	4/19/2007 1:15:49	48	36.9	4/23/2006 1:38:13	26	37
4/14/2008 4:47:05	42	37.3	4/19/2007 1:16:04	46	36.8	4/23/2006 1:38:28	26	37
4/14/2008 4:47:10	41	37.3	4/19/2007 1:16:19	44	36.9	4/23/2006 1:38:43	25	37
4/14/2008 4:47:15	41	37.3	4/19/2007 1:16:34	41	36.8	4/23/2006 1:38:58	27	37
4/14/2008 4:47:20	41	37.3	4/19/2007 1:16:49	39	36.8	4/23/2006 1:39:13	30	37
4/14/2008 4:47:25	40	37.3	4/19/2007 1:17:04	38	36.8	4/23/2006 1:39:28	33	37
4/14/2008 4:47:30	40	37.3	4/19/2007 1:17:19	36	36.9	4/23/2006 1:39:43	36	37
4/14/2008 4:47:35	40	37.3	4/19/2007 1:17:34	35	36.8	4/23/2006 1:39:58	37	37
4/14/2008 4:47:40	40	37.3	4/19/2007 1:17:49	34	36.8	4/23/2006 1:40:13	40	37
4/14/2008 4:47:45	39	37.3	4/19/2007 1:18:04	33	36.8	4/23/2006 1:40:28	44	37
4/14/2008 4:47:50	39	37.3	4/19/2007 1:18:19	32	36.8	4/23/2006 1:40:43	45	37
4/14/2008 4:47:55	38	37.3	4/19/2007 1:18:34	31	36.8	4/23/2006 1:40:58	46	37
4/14/2008 4:48:00	38	37.3	4/19/2007 1:18:49	30	36.8	4/23/2006 1:41:13	48	37
4/14/2008 4:48:05	38	37.3	4/19/2007 1:19:04	29	36.8	4/23/2006 1:41:28	49	37
4/14/2008 4:48:10	38	37.3	4/19/2007 1:19:19	28	36.8	4/23/2006 1:41:43	51	37
4/14/2008 4:48:15	39	37.3	4/19/2007 1:19:34	27	36.8	4/23/2006 1:41:58	49	36.9
4/14/2008 4:48:20	40	37.3	4/19/2007 1:19:49	26	36.8	4/23/2006 1:42:13	46	37
4/14/2008 4:48:25	42	37.4	4/19/2007 1:20:04	26	36.8	4/23/2006 1:42:28	44	36.9
4/14/2008 4:48:30	44	37.4	4/19/2007 1:20:19	25	36.8	4/23/2006 1:42:43	42	36.9
4/14/2008 4:48:35	47	37.4	4/19/2007 1:20:34	25	36.7	4/23/2006 1:42:58	40	36.9
4/14/2008 4:48:40	48	37.4	4/19/2007 1:20:49	24	36.7	4/23/2006 1:43:13	38	36.9
4/14/2008 4:48:45	50	37.4	4/19/2007 1:21:04	23	36.7	4/23/2006 1:43:28	37	36.9
4/14/2008 4:48:50	52	37.4	4/19/2007 1:21:19	23	36.7	4/23/2006 1:43:43	35	36.9
4/14/2008 4:48:55	53	37.4	4/19/2007 1:21:34	22	36.7	4/23/2006 1:43:58	34	36.9
4/14/2008 4:49:00	55	37.4	4/19/2007 1:21:49	22	36.7	4/23/2006 1:44:13	33	36.9
4/14/2008 4:49:05	56	37.4	4/19/2007 1:22:04	21	36.7	4/23/2006 1:44:28	32	36.9
4/14/2008 4:49:10	56	37.4	4/19/2007 1:22:19	21	36.7	4/23/2006 1:44:43	31	36.9
4/14/2008 4:49:15	58	37.4	4/19/2007 1:22:34	20	36.7	4/23/2006 1:44:58	30	36.9
4/14/2008 4:49:20	60	37.4	4/19/2007 1:22:49	21	36.7	4/23/2006 1:45:13	29	36.9
4/14/2008 4:49:25	62	37.4	4/19/2007 1:23:04	21	36.7	4/23/2006 1:45:28	29	36.9
4/14/2008 4:49:30	64	37.4	4/19/2007 1:23:19	22	36.7	4/23/2006 1:45:43	28	36.9
4/14/2008 4:49:35	67	37.4	4/19/2007 1:23:34	25	36.7	4/23/2006 1:45:58	27	36.9
4/14/2008 4:49:40	69	37.4	4/19/2007 1:23:49	27	36.7	4/23/2006 1:46:13	26	36.9
4/14/2008 4:49:45	71	37.4	4/19/2007 1:24:04	29	36.8	4/23/2006 1:46:28	25	36.9
4/14/2008 4:49:50	72	37.4	4/19/2007 1:24:19	32	36.8	4/23/2006 1:46:43	25	36.9
4/14/2008 4:49:55	74	37.4	4/19/2007 1:24:34	35	36.8	4/23/2006 1:46:58	24	36.9
4/14/2008 4:50:00	75	37.4	4/19/2007 1:24:49	37	36.8	4/23/2006 1:47:13	23	37
4/14/2008 4:50:05	76	37.4	4/19/2007 1:25:04	41	36.8	4/23/2006 1:47:28	23	37
4/14/2008 4:50:10	78	37.4	4/19/2007 1:25:19	43	36.8	4/23/2006 1:47:43	24	37
4/14/2008 4:50:15	79	37.4	4/19/2007 1:25:34	47	36.8	4/23/2006 1:47:58	27	37
4/14/2008 4:50:20	81	37.4	4/19/2007 1:25:49	50	36.8	4/23/2006 1:48:13	30	37
4/14/2008 4:50:25	82	37.4	4/19/2007 1:26:04	52	36.8	4/23/2006 1:48:28	33	36.9
4/14/2008 4:50:30	84	37.4	4/19/2007 1:26:19	54	36.8	4/23/2006 1:48:43	36	37
4/14/2008 4:50:35	85	37.4	4/19/2007 1:26:34	56	36.8	4/23/2006 1:48:58	39	36.9
4/14/2008 4:50:40	87	37.4	4/19/2007 1:26:49	58	36.8	4/23/2006 1:49:13	41	37
4/14/2008 4:50:45	88	37.4	4/19/2007 1:27:04	59	36.8	4/23/2006 1:49:28	45	37
4/14/2008 4:50:50	89	37.4	4/19/2007 1:27:19	60	36.8	4/23/2006 1:49:43	47	37
4/14/2008 4:50:55	90	37.4	4/19/2007 1:27:34	61	36.8	4/23/2006 1:49:58	48	37
4/14/2008 4:51:00	90	37.4	4/19/2007 1:27:49	60	36.8	4/23/2006 1:50:13	48	37
4/14/2008 4:51:05	91	37.4	4/19/2007 1:28:04	61	36.8	4/23/2006 1:50:28	50	37

4/14/2008 4:51:10	92	37.4	4/19/2007 1:28:19	61	36.8	4/23/2006 1:50:43	50	37
4/14/2008 4:51:15	93	37.4	4/19/2007 1:28:34	61	36.8	4/23/2006 1:50:58	49	37
4/14/2008 4:51:20	94	37.4	4/19/2007 1:28:49	61	36.8	4/23/2006 1:51:13	49	37
4/14/2008 4:51:25	94	37.4	4/19/2007 1:29:04	62	36.8	4/23/2006 1:51:28	49	37
4/14/2008 4:51:30	95	37.4	4/19/2007 1:29:19	60	36.9	4/23/2006 1:51:43	51	37
4/14/2008 4:51:35	95	37.4	4/19/2007 1:29:34	57	36.9	4/23/2006 1:51:58	51	37
4/14/2008 4:51:40	96	37.4	4/19/2007 1:29:49	54	36.8	4/23/2006 1:52:13	52	37
4/14/2008 4:51:45	96	37.4	4/19/2007 1:30:04	50	36.8	4/23/2006 1:52:28	50	37
4/14/2008 4:51:50	97	37.4	4/19/2007 1:30:19	47	36.9	4/23/2006 1:52:43	50	37
4/14/2008 4:51:55	97	37.4	4/19/2007 1:30:34	44	36.8	4/23/2006 1:52:58	52	36.9
4/14/2008 4:52:00	97	37.4	4/19/2007 1:30:49	42	36.8	4/23/2006 1:53:13	51	37
4/14/2008 4:52:05	97	37.4	4/19/2007 1:31:04	40	36.8	4/23/2006 1:53:28	50	37
4/14/2008 4:52:10	96	37.4	4/19/2007 1:31:19	38	36.8	4/23/2006 1:53:43	52	37
4/14/2008 4:52:15	97	37.4	4/19/2007 1:31:34	37	36.8	4/23/2006 1:53:58	50	37
4/14/2008 4:52:20	97	37.4	4/19/2007 1:31:49	35	36.8	4/23/2006 1:54:13	47	37
4/14/2008 4:52:25	96	37.4	4/19/2007 1:32:04	34	36.8	4/23/2006 1:54:28	49	37
4/14/2008 4:52:30	96	37.4	4/19/2007 1:32:19	33	36.8	4/23/2006 1:54:43	48	37
4/14/2008 4:52:35	95	37.4	4/19/2007 1:32:34	32	36.8	4/23/2006 1:54:58	48	36.9
4/14/2008 4:52:40	96	37.4	4/19/2007 1:32:49	31	36.8	4/23/2006 1:55:13	48	36.9
4/14/2008 4:52:45	97	37.4	4/19/2007 1:33:04	30	36.8	4/23/2006 1:55:28	47	36.9
4/14/2008 4:52:50	97	37.4	4/19/2007 1:33:19	29	36.8	4/23/2006 1:55:43	47	36.9
4/14/2008 4:52:55	97	37.4	4/19/2007 1:33:34	28	36.8	4/23/2006 1:55:58	47	36.9
4/14/2008 4:53:00	98	37.4	4/19/2007 1:33:49	28	36.8	4/23/2006 1:56:13	45	36.8
4/14/2008 4:53:05	99	37.4	4/19/2007 1:34:04	27	36.8	4/23/2006 1:56:28	43	36.8
4/14/2008 4:53:10	98	37.4	4/19/2007 1:34:19	27	36.8	4/23/2006 1:56:43	42	36.8
4/14/2008 4:53:15	98	37.4	4/19/2007 1:34:34	28	36.8	4/23/2006 1:56:58	41	36.8
4/14/2008 4:53:20	97	37.4	4/19/2007 1:34:49	29	36.8	4/23/2006 1:57:13	39	36.8
4/14/2008 4:53:25	97	37.4	4/19/2007 1:35:04	31	36.8	4/23/2006 1:57:28	38	36.8
4/14/2008 4:53:30	96	37.4	4/19/2007 1:35:19	33	36.8	4/23/2006 1:57:43	37	36.8
4/14/2008 4:53:35	95	37.4	4/19/2007 1:35:34	36	36.8	4/23/2006 1:57:58	36	36.8
4/14/2008 4:53:40	94	37.4	4/19/2007 1:35:49	38	36.8	4/23/2006 1:58:13	35	36.8
4/14/2008 4:53:45	93	37.4	4/19/2007 1:36:04	40	36.8	4/23/2006 1:58:28	35	36.8
4/14/2008 4:53:50	93	37.4	4/19/2007 1:36:19	43	36.8	4/23/2006 1:58:43	34	36.8
4/14/2008 4:53:55	93	37.4	4/19/2007 1:36:34	45	36.8	4/23/2006 1:58:58	33	36.8
4/14/2008 4:54:00	92	37.4	4/19/2007 1:36:49	47	36.8	4/23/2006 1:59:13	32	36.8
4/14/2008 4:54:05	101	37.4	4/19/2007 1:37:04	49	36.8	4/23/2006 1:59:28	31	37
4/14/2008 4:54:10	92	37.4	4/19/2007 1:37:19	52	36.8	4/23/2006 1:59:43	29	37
4/14/2008 4:54:15	92	37.4	4/19/2007 1:37:34	54	36.8	4/23/2006 1:59:58	26	37
4/14/2008 4:54:20	92	37.4	4/19/2007 1:37:49	56	36.8	4/23/2006 2:00:13	26	37
4/14/2008 4:54:25	91	37.4	4/19/2007 1:38:04	57	36.8	4/23/2006 2:00:28	27	37
4/14/2008 4:54:30	91	37.4	4/19/2007 1:38:19	58	36.8	4/23/2006 2:00:43	30	37
4/14/2008 4:54:35	90	37.4	4/19/2007 1:38:34	60	36.8	4/23/2006 2:00:58	32	37
4/14/2008 4:54:40	90	37.4	4/19/2007 1:38:49	62	36.8	4/23/2006 2:01:13	35	37
4/14/2008 4:54:45	90	37.4	4/19/2007 1:39:04	61	36.8	4/23/2006 2:01:28	38	37
4/14/2008 4:54:50	90	37.4	4/19/2007 1:39:19	62	36.8	4/23/2006 2:01:43	39	37
4/14/2008 4:54:55	90	37.4	4/19/2007 1:39:34	62	36.8	4/23/2006 2:01:58	40	37
4/14/2008 4:55:00	90	37.4	4/19/2007 1:39:49	61	36.8	4/23/2006 2:02:13	44	37
4/14/2008 4:55:05	90	37.4	4/19/2007 1:40:04	59	36.8	4/23/2006 2:02:28	44	37
4/14/2008 4:55:10	90	37.4	4/19/2007 1:40:19	55	36.8	4/23/2006 2:02:43	47	37
4/14/2008 4:55:15	90	37.4	4/19/2007 1:40:34	52	36.8	4/23/2006 2:02:58	46	37
4/14/2008 4:55:20	90	37.4	4/19/2007 1:40:49	48	36.8	4/23/2006 2:03:13	48	37
4/14/2008 4:55:25	90	37.4	4/19/2007 1:41:04	45	36.8	4/23/2006 2:03:28	50	36.9
4/14/2008 4:55:30	90	37.4	4/19/2007 1:41:19	43	36.9	4/23/2006 2:03:43	50	36.9
4/14/2008 4:55:35	90	37.4	4/19/2007 1:41:34	41	36.8	4/23/2006 2:03:58	49	37
4/14/2008 4:55:40	89	37.4	4/19/2007 1:41:49	39	36.8	4/23/2006 2:04:13	49	36.9
4/14/2008 4:55:45	89	37.4	4/19/2007 1:42:04	37	36.8	4/23/2006 2:04:28	49	36.9
4/14/2008 4:55:50	89	37.4	4/19/2007 1:42:19	35	36.8	4/23/2006 2:04:43	48	37
4/14/2008 4:55:55	90	37.4	4/19/2007 1:42:34	34	36.8	4/23/2006 2:04:58	47	36.9
4/14/2008 4:56:00	90	37.4	4/19/2007 1:42:49	34	36.8	4/23/2006 2:05:13	47	36.9
4/14/2008 4:56:05	89	37.4	4/19/2007 1:43:04	33	36.8	4/23/2006 2:05:28	46	36.9
4/14/2008 4:56:10	88	37.4	4/19/2007 1:43:19	31	36.8	4/23/2006 2:05:43	46	36.8
4/14/2008 4:56:15	86	37.4	4/19/2007 1:43:34	30	36.8	4/23/2006 2:05:58	46	36.8
4/14/2008 4:56:20	84	37.4	4/19/2007 1:43:49	30	36.8	4/23/2006 2:06:13	45	36.8
4/14/2008 4:56:25	81	37.4	4/19/2007 1:44:04	29	36.8	4/23/2006 2:06:28	45	36.8
4/14/2008 4:56:30	79	37.4	4/19/2007 1:44:19	28	36.8	4/23/2006 2:06:43	45	36.8
4/14/2008 4:56:35	76	37.4	4/19/2007 1:44:34	27	36.8	4/23/2006 2:06:58	43	36.8
4/14/2008 4:56:40	74	37.4	4/19/2007 1:44:49	27	36.7	4/23/2006 2:07:13	41	36.8
4/14/2008 4:56:45	72	37.4	4/19/2007 1:45:04	26	36.7	4/23/2006 2:07:28	40	36.8
4/14/2008 4:56:50	70	37.3	4/19/2007 1:45:19	25	36.8	4/23/2006 2:07:43	38	36.8
4/14/2008 4:56:55	68	37.3	4/19/2007 1:45:34	25	36.7	4/23/2006 2:07:58	37	36.8
4/14/2008 4:57:00	67	37.3	4/19/2007 1:45:49	24	36.7	4/23/2006 2:08:13	35	36.8
4/14/2008 4:57:05	65	37.3	4/19/2007 1:46:04	24	36.7	4/23/2006 2:08:28	34	36.7
4/14/2008 4:57:10	64	37.3	4/19/2007 1:46:19	23	36.7	4/23/2006 2:08:43	33	36.8
4/14/2008 4:57:15	63	37.3	4/19/2007 1:46:34	23	36.7	4/23/2006 2:08:58	32	36.7
4/14/2008 4:57:20	62	37.3	4/19/2007 1:46:49	22	36.7	4/23/2006 2:09:13	31	36.8
4/14/2008 4:57:25	61	37.3	4/19/2007 1:47:04	23	36.7	4/23/2006 2:09:28	31	36.8
4/14/2008 4:57:30	60	37.3	4/19/2007 1:47:19	23	36.7	4/23/2006 2:09:43	29	36.8
4/14/2008 4:57:35	59	37.3	4/19/2007 1:47:34	24	36.7	4/23/2006 2:09:58	29	36.7
4/14/2008 4:57:40	58	37.3	4/19/2007 1:47:49	27	36.7	4/23/2006 2:10:13	28	36.7
4/14/2008 4:57:45	57	37.3	4/19/2007 1:48:04	29	36.7	4/23/2006 2:10:28	27	36.8
4/14/2008 4:57:50	57	37.3	4/19/2007 1:48:19	31	36.7	4/23/2006 2:10:43	26	36.9
4/14/2008 4:57:55	56	37.3	4/19/2007 1:48:34	35	36.7	4/23/2006 2:10:58	23	37
4/14/2008 4:58:00	55	37.3	4/19/2007 1:48:49	37	36.8	4/23/2006 2:11:13	24	36.9
4/14/2008 4:58:05	54	37.3	4/19/2007 1:49:04	40	36.8	4/23/2006 2:11:28	25	36.9
4/14/2008 4:58:10	53	37.3	4/19/2007 1:49:19	43	36.8	4/23/2006 2:11:43	26	37
4/14/2008 4:58:15	53	37.3	4/19/2007 1:49:34	47	36.8	4/23/2006 2:11:58	29	37
4/14/2008 4:58:20	52	37.3	4/19/2007 1:49:49	50	36.8	4/23/2006 2:12:13	31	37

4/14/2008 4:58:25	52	37.3	4/19/2007 1:50:04	53	36.8	4/23/2006 2:12:28	34	36.9
4/14/2008 4:58:30	51	37.3	4/19/2007 1:50:19	55	36.8	4/23/2006 2:12:43	37	37
4/14/2008 4:58:35	51	37.3	4/19/2007 1:50:34	57	36.8	4/23/2006 2:12:58	40	36.9
4/14/2008 4:58:40	50	37.3	4/19/2007 1:50:49	59	36.8	4/23/2006 2:13:13	41	37
4/14/2008 4:58:45	50	37.3	4/19/2007 1:51:04	60	36.8	4/23/2006 2:13:28	43	36.9
4/14/2008 4:58:50	49	37.3	4/19/2007 1:51:19	62	36.8	4/23/2006 2:13:43	47	36.9
4/14/2008 4:58:55	48	37.3	4/19/2007 1:51:34	62	36.8	4/23/2006 2:13:58	48	36.9
4/14/2008 4:59:00	48	37.3	4/19/2007 1:51:49	63	36.8	4/23/2006 2:14:13	49	36.9
4/14/2008 4:59:05	48	37.3	4/19/2007 1:52:04	63	36.8	4/23/2006 2:14:28	49	37
4/14/2008 4:59:10	47	37.3	4/19/2007 1:52:19	63	36.8	4/23/2006 2:14:43	48	36.9
4/14/2008 4:59:15	47	37.3	4/19/2007 1:52:34	63	36.8	4/23/2006 2:14:58	49	37
4/14/2008 4:59:20	47	37.3	4/19/2007 1:52:49	64	36.8	4/23/2006 2:15:13	48	37
4/14/2008 4:59:25	46	37.3	4/19/2007 1:53:04	63	36.9	4/23/2006 2:15:28	49	37
4/14/2008 4:59:30	46	37.3	4/19/2007 1:53:19	63	36.9	4/23/2006 2:15:43	49	37
4/14/2008 4:59:35	45	37.2	4/19/2007 1:53:34	63	36.9	4/23/2006 2:15:58	47	37
4/14/2008 4:59:40	45	37.2	4/19/2007 1:53:49	64	36.9	4/23/2006 2:16:13	45	37
4/14/2008 4:59:45	44	37.2	4/19/2007 1:54:04	63	36.9	4/23/2006 2:16:28	46	36.9
4/14/2008 4:59:50	44	37.2	4/19/2007 1:54:19	63	36.9	4/23/2006 2:16:43	46	37
4/14/2008 4:59:55	44	37.2	4/19/2007 1:54:34	62	36.9	4/23/2006 2:16:58	45	37
4/14/2008 5:00:00	43	37.2	4/19/2007 1:54:49	61	36.9	4/23/2006 2:17:13	44	37
4/14/2008 5:00:05	43	37.2	4/19/2007 1:55:04	60	36.9	4/23/2006 2:17:28	46	37
4/14/2008 5:00:10	42	37.2	4/19/2007 1:55:19	59	36.9	4/23/2006 2:17:43	45	36.9
4/14/2008 5:00:15	42	37.2	4/19/2007 1:55:34	59	36.9	4/23/2006 2:17:58	45	36.9
4/14/2008 5:00:20	42	37.2	4/19/2007 1:55:49	59	36.9	4/23/2006 2:18:13	44	36.9
4/14/2008 5:00:25	42	37.2	4/19/2007 1:56:04	58	36.9	4/23/2006 2:18:28	45	36.9
4/14/2008 5:00:30	41	37.2	4/19/2007 1:56:19	57	36.9	4/23/2006 2:18:43	45	36.8
4/14/2008 5:00:35	41	37.2	4/19/2007 1:56:34	55	36.9	4/23/2006 2:18:58	45	36.8
4/14/2008 5:00:40	41	37.2	4/19/2007 1:56:49	52	36.9	4/23/2006 2:19:13	44	36.8
4/14/2008 5:00:45	40	37.2	4/19/2007 1:57:04	49	36.9	4/23/2006 2:19:28	43	36.8
4/14/2008 5:00:50	40	37.2	4/19/2007 1:57:19	46	36.9	4/23/2006 2:19:43	41	36.8
4/14/2008 5:00:55	40	37.2	4/19/2007 1:57:34	44	36.9	4/23/2006 2:19:58	39	36.8
4/14/2008 5:01:00	39	37.2	4/19/2007 1:57:49	41	36.9	4/23/2006 2:20:13	37	36.8
4/14/2008 5:01:05	39	37.2	4/19/2007 1:58:04	39	36.9	4/23/2006 2:20:28	35	36.8
4/14/2008 5:01:10	39	37.2	4/19/2007 1:58:19	38	36.9	4/23/2006 2:20:43	34	36.8
4/14/2008 5:01:15	39	37.2	4/19/2007 1:58:34	36	36.9	4/23/2006 2:20:58	33	36.8
4/14/2008 5:01:20	38	37.2	4/19/2007 1:58:49	35	36.8	4/23/2006 2:21:13	31	36.8
4/14/2008 5:01:25	38	37.2	4/19/2007 1:59:04	33	36.8	4/23/2006 2:21:28	31	36.8
4/14/2008 5:01:30	38	37.2	4/19/2007 1:59:19	33	36.8	4/23/2006 2:21:43	30	36.8
4/14/2008 5:01:35	37	37.2	4/19/2007 1:59:34	32	36.8	4/23/2006 2:21:58	29	36.8
4/14/2008 5:01:40	37	37.2	4/19/2007 1:59:49	31	36.8	4/23/2006 2:22:13	28	36.8
4/14/2008 5:01:45	37	37.2	4/19/2007 2:00:04	29	36.8	4/23/2006 2:22:28	28	36.8
4/14/2008 5:01:50	36	37.2	4/19/2007 2:00:19	29	36.8	4/23/2006 2:22:43	27	36.8
4/14/2008 5:01:55	36	37.2	4/19/2007 2:00:34	28	36.8	4/23/2006 2:22:58	26	36.8
4/14/2008 5:02:00	36	37.2	4/19/2007 2:00:49	27	36.8	4/23/2006 2:23:13	25	36.8
4/14/2008 5:02:05	36	37.2	4/19/2007 2:01:04	26	36.8	4/23/2006 2:23:28	25	36.8
4/14/2008 5:02:10	35	37.2	4/19/2007 2:01:19	26	36.8	4/23/2006 2:23:43	24	36.8
4/14/2008 5:02:15	35	37.2	4/19/2007 2:01:34	25	36.8	4/23/2006 2:23:58	23	36.8
4/14/2008 5:02:20	36	37.2	4/19/2007 2:01:49	24	36.8	4/23/2006 2:24:13	22	36.8
4/14/2008 5:02:25	37	37.3	4/19/2007 2:02:04	24	36.8	4/23/2006 2:24:28	22	36.8
4/14/2008 5:02:30	39	37.3	4/19/2007 2:02:19	23	36.8	4/23/2006 2:24:43	21	36.9
4/14/2008 5:02:35	42	37.3	4/19/2007 2:02:34	25	36.8	4/23/2006 2:24:58	20	36.9
4/14/2008 5:02:40	43	37.3	4/19/2007 2:02:49	26	36.8	4/23/2006 2:25:13	20	36.9
4/14/2008 5:02:45	44	37.3	4/19/2007 2:03:04	28	36.8	4/23/2006 2:25:28	22	37
4/14/2008 5:02:50	46	37.3	4/19/2007 2:03:19	30	36.8	4/23/2006 2:25:43	24	37
4/14/2008 5:02:55	47	37.3	4/19/2007 2:03:34	32	36.8	4/23/2006 2:25:58	27	36.9
4/14/2008 5:03:00	48	37.3	4/19/2007 2:03:49	35	36.8	4/23/2006 2:26:13	30	37
4/14/2008 5:03:05	50	37.3	4/19/2007 2:04:04	39	36.8	4/23/2006 2:26:28	35	37
4/14/2008 5:03:10	51	37.3	4/19/2007 2:04:19	43	36.8	4/23/2006 2:26:43	39	37
4/14/2008 5:03:15	53	37.3	4/19/2007 2:04:34	46	36.8	4/23/2006 2:26:58	42	37
4/14/2008 5:03:20	54	37.3	4/19/2007 2:04:49	49	36.8	4/23/2006 2:27:13	45	36.9
4/14/2008 5:03:25	55	37.3	4/19/2007 2:05:04	52	36.8	4/23/2006 2:27:28	45	37
4/14/2008 5:03:30	57	37.3	4/19/2007 2:05:19	55	36.9	4/23/2006 2:27:43	48	36.9
4/14/2008 5:03:35	58	37.3	4/19/2007 2:05:34	58	36.9	4/23/2006 2:27:58	50	37
4/14/2008 5:03:40	59	37.3	4/19/2007 2:05:49	60	36.9	4/23/2006 2:28:13	50	37
4/14/2008 5:03:45	61	37.3	4/19/2007 2:06:04	60	36.9	4/23/2006 2:28:28	50	36.9
4/14/2008 5:03:50	63	37.3	4/19/2007 2:06:19	60	36.9	4/23/2006 2:28:43	50	36.9
4/14/2008 5:03:55	64	37.3	4/19/2007 2:06:34	58	36.9	4/23/2006 2:28:58	51	37
4/14/2008 5:04:00	66	37.3	4/19/2007 2:06:49	55	36.9	4/23/2006 2:29:13	51	37
4/14/2008 5:04:05	68	37.3	4/19/2007 2:07:04	51	36.9	4/23/2006 2:29:28	51	36.9
4/14/2008 5:04:10	69	37.3	4/19/2007 2:07:19	48	36.9	4/23/2006 2:29:43	51	37
4/14/2008 5:04:15	71	37.3	4/19/2007 2:07:34	45	36.9	4/23/2006 2:29:58	50	36.9
4/14/2008 5:04:20	72	37.3	4/19/2007 2:07:49	43	36.9	4/23/2006 2:30:13	49	36.9
4/14/2008 5:04:25	73	37.3	4/19/2007 2:08:04	41	36.9	4/23/2006 2:30:28	49	36.9
4/14/2008 5:04:30	75	37.3	4/19/2007 2:08:19	39	36.9	4/23/2006 2:30:43	49	36.9
4/14/2008 5:04:35	76	37.3	4/19/2007 2:08:34	38	36.9	4/23/2006 2:30:58	47	36.9
4/14/2008 5:04:40	78	37.3	4/19/2007 2:08:49	36	36.9	4/23/2006 2:31:13	45	36.8
4/14/2008 5:04:45	79	37.3	4/19/2007 2:09:04	35	36.8	4/23/2006 2:31:28	42	36.8
4/14/2008 5:04:50	81	37.3	4/19/2007 2:09:19	33	36.9	4/23/2006 2:31:43	40	36.8
4/14/2008 5:04:55	82	37.3	4/19/2007 2:09:34	32	36.9	4/23/2006 2:31:58	38	36.8
4/14/2008 5:05:00	83	37.3	4/19/2007 2:09:49	32	36.8	4/23/2006 2:32:13	37	36.8
4/14/2008 5:05:05	84	37.3	4/19/2007 2:10:04	31	36.8	4/23/2006 2:32:28	35	36.8
4/14/2008 5:05:10	85	37.3	4/19/2007 2:10:19	29	36.8	4/23/2006 2:32:43	34	36.8
4/14/2008 5:05:15	86	37.3	4/19/2007 2:10:34	29	36.8	4/23/2006 2:32:58	33	36.8
4/14/2008 5:05:20	87	37.3	4/19/2007 2:10:49	28	36.8	4/23/2006 2:33:13	32	36.8
4/14/2008 5:05:25	88	37.3	4/19/2007 2:11:04	27	36.8	4/23/2006 2:33:28	31	36.8
4/14/2008 5:05:30	88	37.3	4/19/2007 2:11:19	26	36.8	4/23/2006 2:33:43	30	36.8
4/14/2008 5:05:35	89	37.3	4/19/2007 2:11:34	26	36.8	4/23/2006 2:33:58	29	36.8

4/14/2008 5:05:40	90	37.3	4/19/2007 2:11:49	25	36.8	4/23/2006 2:34:13	28	36.8
4/14/2008 5:05:45	90	37.3	4/19/2007 2:12:04	25	36.8	4/23/2006 2:34:28	28	36.8
4/14/2008 5:05:50	91	37.3	4/19/2007 2:12:19	24	36.8	4/23/2006 2:34:43	28	36.8
4/14/2008 5:05:55	90	37.3	4/19/2007 2:12:34	23	36.7	4/23/2006 2:34:58	26	36.8
4/14/2008 5:06:00	91	37.3	4/19/2007 2:12:49	22	36.7	4/23/2006 2:35:13	26	36.8
4/14/2008 5:06:05	91	37.3	4/19/2007 2:13:04	22	36.7	4/23/2006 2:35:28	25	36.8
4/14/2008 5:06:10	91	37.3	4/19/2007 2:13:19	22	36.8	4/23/2006 2:35:43	25	36.8
4/14/2008 5:06:15	91	37.3	4/19/2007 2:13:34	24	36.8	4/23/2006 2:35:58	24	36.8
4/14/2008 5:06:20	91	37.3	4/19/2007 2:13:49	26	36.8	4/23/2006 2:36:13	24	36.8
4/14/2008 5:06:25	91	37.3	4/19/2007 2:14:04	28	36.8	4/23/2006 2:36:28	22	36.8
4/14/2008 5:06:30	91	37.3	4/19/2007 2:14:19	31	36.8	4/23/2006 2:36:43	22	36.9
4/14/2008 5:06:35	91	37.3	4/19/2007 2:14:34	34	36.8	4/23/2006 2:36:58	21	36.9
4/14/2008 5:06:40	90	37.3	4/19/2007 2:14:49	36	36.8	4/23/2006 2:37:13	22	37
4/14/2008 5:06:45	89	37.3	4/19/2007 2:15:04	40	36.8	4/23/2006 2:37:28	24	37
4/14/2008 5:06:50	87	37.3	4/19/2007 2:15:19	43	36.8	4/23/2006 2:37:43	26	37
4/14/2008 5:06:55	85	37.3	4/19/2007 2:15:34	47	36.8	4/23/2006 2:37:58	29	36.9
4/14/2008 5:07:00	82	37.3	4/19/2007 2:15:49	50	36.8	4/23/2006 2:38:13	32	37
4/14/2008 5:07:05	80	37.3	4/19/2007 2:16:04	53	36.8	4/23/2006 2:38:28	36	37
4/14/2008 5:07:10	78	37.3	4/19/2007 2:16:19	56	36.8	4/23/2006 2:38:43	39	37
4/14/2008 5:07:15	75	37.3	4/19/2007 2:16:34	58	36.8	4/23/2006 2:38:58	42	37
4/14/2008 5:07:20	73	37.3	4/19/2007 2:16:49	60	36.8	4/23/2006 2:39:13	45	36.9
4/14/2008 5:07:25	71	37.3	4/19/2007 2:17:04	61	36.8	4/23/2006 2:39:28	47	36.9
4/14/2008 5:07:30	70	37.3	4/19/2007 2:17:19	62	36.9	4/23/2006 2:39:43	49	37
4/14/2008 5:07:35	68	37.3	4/19/2007 2:17:34	63	36.8	4/23/2006 2:39:58	50	37
4/14/2008 5:07:40	67	37.3	4/19/2007 2:17:49	64	36.8	4/23/2006 2:40:13	51	37
4/14/2008 5:07:45	65	37.3	4/19/2007 2:18:04	64	36.9	4/23/2006 2:40:28	52	36.9
4/14/2008 5:07:50	64	37.3	4/19/2007 2:18:19	64	36.9	4/23/2006 2:40:43	52	37
4/14/2008 5:07:55	63	37.3	4/19/2007 2:18:34	64	36.9	4/23/2006 2:40:58	52	36.9
4/14/2008 5:08:00	62	37.3	4/19/2007 2:18:49	63	36.9	4/23/2006 2:41:13	52	37
4/14/2008 5:08:05	61	37.3	4/19/2007 2:19:04	62	36.9	4/23/2006 2:41:28	52	37
4/14/2008 5:08:10	59	37.3	4/19/2007 2:19:19	61	36.9	4/23/2006 2:41:43	52	37
4/14/2008 5:08:15	59	37.3	4/19/2007 2:19:34	58	36.9	4/23/2006 2:41:58	52	36.9
4/14/2008 5:08:20	58	37.3	4/19/2007 2:19:49	54	36.9	4/23/2006 2:42:13	52	36.9
4/14/2008 5:08:25	57	37.3	4/19/2007 2:20:04	51	36.9	4/23/2006 2:42:28	51	36.9
4/14/2008 5:08:30	56	37.3	4/19/2007 2:20:19	48	36.9	4/23/2006 2:42:43	51	37
4/14/2008 5:08:35	56	37.3	4/19/2007 2:20:34	45	36.9	4/23/2006 2:42:58	50	37
4/14/2008 5:08:40	55	37.3	4/19/2007 2:20:49	42	36.9	4/23/2006 2:43:13	50	36.9
4/14/2008 5:08:45	54	37.3	4/19/2007 2:21:04	40	36.8	4/23/2006 2:43:28	50	36.9
4/14/2008 5:08:50	54	37.3	4/19/2007 2:21:19	38	36.9	4/23/2006 2:43:43	49	36.8
4/14/2008 5:08:55	53	37.2	4/19/2007 2:21:34	37	36.8	4/23/2006 2:43:58	50	36.9
4/14/2008 5:09:00	52	37.2	4/19/2007 2:21:49	35	36.8	4/23/2006 2:44:13	49	36.9
4/14/2008 5:09:05	52	37.2	4/19/2007 2:22:04	34	36.8	4/23/2006 2:44:28	46	36.9
4/14/2008 5:09:10	51	37.2	4/19/2007 2:22:19	33	36.8	4/23/2006 2:44:43	45	36.9
4/14/2008 5:09:15	51	37.2	4/19/2007 2:22:34	32	36.8	4/23/2006 2:44:58	45	36.9
4/14/2008 5:09:20	50	37.2	4/19/2007 2:22:49	31	36.8	4/23/2006 2:45:13	45	36.8
4/14/2008 5:09:25	50	37.2	4/19/2007 2:23:04	30	36.8	4/23/2006 2:45:28	46	36.8
4/14/2008 5:09:30	49	37.2	4/19/2007 2:23:19	29	36.8	4/23/2006 2:45:43	45	36.8
4/14/2008 5:09:35	49	37.2	4/19/2007 2:23:34	28	36.8	4/23/2006 2:45:58	43	36.7
4/14/2008 5:09:40	48	37.3	4/19/2007 2:23:49	28	36.8	4/23/2006 2:46:13	41	36.8
4/14/2008 5:09:45	48	37.2	4/19/2007 2:24:04	27	36.8	4/23/2006 2:46:28	39	36.8
4/14/2008 5:09:50	47	37.2	4/19/2007 2:24:19	26	36.8	4/23/2006 2:46:43	37	36.8
4/14/2008 5:09:55	47	37.2	4/19/2007 2:24:34	25	36.7	4/23/2006 2:46:58	36	36.8
4/14/2008 5:10:00	47	37.2	4/19/2007 2:24:49	24	36.7	4/23/2006 2:47:13	35	36.7
4/14/2008 5:10:05	46	37.2	4/19/2007 2:25:04	24	36.8	4/23/2006 2:47:28	34	36.7
4/14/2008 5:10:10	46	37.2	4/19/2007 2:25:19	24	36.8	4/23/2006 2:47:43	33	36.7
4/14/2008 5:10:15	45	37.2	4/19/2007 2:25:34	26	36.8	4/23/2006 2:47:58	32	36.8
4/14/2008 5:10:20	45	37.2	4/19/2007 2:25:49	28	36.8	4/23/2006 2:48:13	31	36.7
4/14/2008 5:10:25	45	37.2	4/19/2007 2:26:04	30	36.8	4/23/2006 2:48:28	30	36.7
4/14/2008 5:10:30	44	37.2	4/19/2007 2:26:19	31	36.8	4/23/2006 2:48:43	29	36.7
4/14/2008 5:10:35	44	37.2	4/19/2007 2:26:34	33	36.8	4/23/2006 2:48:58	28	36.7
4/14/2008 5:10:40	44	37.2	4/19/2007 2:26:49	36	36.8	4/23/2006 2:49:13	27	36.7
4/14/2008 5:10:45	44	37.2	4/19/2007 2:27:04	40	36.8	4/23/2006 2:49:28	27	36.7
4/14/2008 5:10:50	45	37.3	4/19/2007 2:27:19	42	36.8	4/23/2006 2:49:43	26	36.8
4/14/2008 5:10:55	47	37.3	4/19/2007 2:27:34	46	36.8	4/23/2006 2:49:58	26	36.7
4/14/2008 5:11:00	49	37.3	4/19/2007 2:27:49	49	36.8	4/23/2006 2:50:13	25	36.8
4/14/2008 5:11:05	51	37.3	4/19/2007 2:28:04	53	36.8	4/23/2006 2:50:28	24	36.9
4/14/2008 5:11:10	53	37.3	4/19/2007 2:28:19	55	36.9	4/23/2006 2:50:43	23	37
4/14/2008 5:11:15	54	37.3	4/19/2007 2:28:34	57	36.8	4/23/2006 2:50:58	23	36.9
4/14/2008 5:11:20	56	37.3	4/19/2007 2:28:49	59	36.9	4/23/2006 2:51:13	24	36.9
4/14/2008 5:11:25	57	37.3	4/19/2007 2:29:04	60	36.9	4/23/2006 2:51:28	26	36.9
4/14/2008 5:11:30	58	37.3	4/19/2007 2:29:19	59	36.9	4/23/2006 2:51:43	29	37
4/14/2008 5:11:35	59	37.2	4/19/2007 2:29:34	57	36.9	4/23/2006 2:51:58	32	37
4/14/2008 5:11:40	59	37.3	4/19/2007 2:29:49	53	36.9	4/23/2006 2:52:13	36	36.9
4/14/2008 5:11:45	60	37.3	4/19/2007 2:30:04	50	36.9	4/23/2006 2:52:28	39	37
4/14/2008 5:11:50	61	37.3	4/19/2007 2:30:19	47	36.9	4/23/2006 2:52:43	41	36.9
4/14/2008 5:11:55	63	37.3	4/19/2007 2:30:34	44	36.9	4/23/2006 2:52:58	44	36.9
4/14/2008 5:12:00	65	37.3	4/19/2007 2:30:49	43	36.9	4/23/2006 2:53:13	45	37
4/14/2008 5:12:05	66	37.3	4/19/2007 2:31:04	40	36.9	4/23/2006 2:53:28	46	36.9
4/14/2008 5:12:10	68	37.3	4/19/2007 2:31:19	39	36.9	4/23/2006 2:53:43	47	37
4/14/2008 5:12:15	68	37.3	4/19/2007 2:31:34	37	36.9	4/23/2006 2:53:58	48	36.9
4/14/2008 5:12:20	70	37.3	4/19/2007 2:31:49	35	36.8	4/23/2006 2:54:13	49	36.8
4/14/2008 5:12:25	70	37.3	4/19/2007 2:32:04	34	36.8	4/23/2006 2:54:28	50	36.8
4/14/2008 5:12:30	70	37.3	4/19/2007 2:32:19	33	36.8	4/23/2006 2:54:43	50	36.8
4/14/2008 5:12:35	71	37.3	4/19/2007 2:32:34	32	36.8	4/23/2006 2:54:58	49	36.8
4/14/2008 5:12:40	73	37.3	4/19/2007 2:32:49	31	36.8	4/23/2006 2:55:13	46	36.7
4/14/2008 5:12:45	74	37.3	4/19/2007 2:33:04	31	36.8	4/23/2006 2:55:28	43	36.7
4/14/2008 5:12:50	75	37.3	4/19/2007 2:33:19	29	36.8	4/23/2006 2:55:43	41	36.7

4/14/2008 5:12:55	77	37.3	4/19/2007 2:33:34	29	36.8	4/23/2006 2:55:58	39	36.7
4/14/2008 5:13:00	77	37.3	4/19/2007 2:33:49	28	36.8	4/23/2006 2:56:13	38	36.7
4/14/2008 5:13:05	78	37.3	4/19/2007 2:34:04	28	36.8	4/23/2006 2:56:28	36	36.7
4/14/2008 5:13:10	79	37.3	4/19/2007 2:34:19	27	36.8	4/23/2006 2:56:43	35	36.7
4/14/2008 5:13:15	80	37.3	4/19/2007 2:34:34	27	36.8	4/23/2006 2:56:58	34	36.7
4/14/2008 5:13:20	81	37.3	4/19/2007 2:34:49	26	36.7	4/23/2006 2:57:13	33	36.7
4/14/2008 5:13:25	82	37.3	4/19/2007 2:35:04	25	36.7	4/23/2006 2:57:28	32	36.7
4/14/2008 5:13:30	83	37.3	4/19/2007 2:35:19	25	36.7	4/23/2006 2:57:43	31	36.7
4/14/2008 5:13:35	83	37.3	4/19/2007 2:35:34	24	36.7	4/23/2006 2:57:58	30	36.7
4/14/2008 5:13:40	84	37.3	4/19/2007 2:35:49	23	36.7	4/23/2006 2:58:13	29	36.7
4/14/2008 5:13:45	84	37.3	4/19/2007 2:36:04	23	36.7	4/23/2006 2:58:28	29	36.7
4/14/2008 5:13:50	85	37.3	4/19/2007 2:36:19	22	36.7	4/23/2006 2:58:43	28	36.7
4/14/2008 5:13:55	86	37.3	4/19/2007 2:36:34	22	36.7	4/23/2006 2:58:58	27	36.7
4/14/2008 5:14:00	86	37.3	4/19/2007 2:36:49	24	36.7	4/23/2006 2:59:13	27	36.7
4/14/2008 5:14:05	87	37.3	4/19/2007 2:37:04	25	36.7	4/23/2006 2:59:28	26	36.7
4/14/2008 5:14:10	88	37.3	4/19/2007 2:37:19	27	36.8	4/23/2006 2:59:43	25	36.7
4/14/2008 5:14:15	89	37.3	4/19/2007 2:37:34	30	36.8	4/23/2006 2:59:58	25	36.7
4/14/2008 5:14:20	90	37.3	4/19/2007 2:37:49	33	36.8	4/23/2006 3:00:13	24	36.7
4/14/2008 5:14:25	90	37.3	4/19/2007 2:38:04	36	36.8	4/23/2006 3:00:28	23	36.9
4/14/2008 5:14:30	91	37.3	4/19/2007 2:38:19	40	36.8	4/23/2006 3:00:43	23	36.9
4/14/2008 5:14:35	92	37.3	4/19/2007 2:38:34	42	36.8	4/23/2006 3:00:58	23	36.9
4/14/2008 5:14:40	92	37.3	4/19/2007 2:38:49	45	36.8	4/23/2006 3:01:13	25	36.9
4/14/2008 5:14:45	93	37.3	4/19/2007 2:39:04	47	36.8	4/23/2006 3:01:28	28	36.9
4/14/2008 5:14:50	93	37.3	4/19/2007 2:39:19	50	36.8	4/23/2006 3:01:43	30	36.9
4/14/2008 5:14:55	94	37.3	4/19/2007 2:39:34	54	36.8	4/23/2006 3:01:58	33	36.9
4/14/2008 5:15:00	93	37.3	4/19/2007 2:39:49	56	36.8	4/23/2006 3:02:13	36	36.9
4/14/2008 5:15:05	94	37.3	4/19/2007 2:40:04	59	36.8	4/23/2006 3:02:28	39	36.9
4/14/2008 5:15:10	93	37.3	4/19/2007 2:40:19	61	36.8	4/23/2006 3:02:43	41	36.9
4/14/2008 5:15:15	93	37.3	4/19/2007 2:40:34	62	36.8	4/23/2006 3:02:58	43	36.8
4/14/2008 5:15:20	93	37.3	4/19/2007 2:40:49	64	36.8	4/23/2006 3:03:13	45	36.9
4/14/2008 5:15:25	92	37.3	4/19/2007 2:41:04	64	36.8	4/23/2006 3:03:28	45	36.8
4/14/2008 5:15:30	91	37.3	4/19/2007 2:41:19	65	36.9	4/23/2006 3:03:43	45	36.7
4/14/2008 5:15:35	90	37.3	4/19/2007 2:41:34	65	36.9	4/23/2006 3:03:58	46	36.8
4/14/2008 5:15:40	89	37.2	4/19/2007 2:41:49	65	36.9	4/23/2006 3:04:13	46	36.7
4/14/2008 5:15:45	88	37.3	4/19/2007 2:42:04	64	36.9	4/23/2006 3:04:28	43	36.7
4/14/2008 5:15:50	87	37.3	4/19/2007 2:42:19	64	36.9	4/23/2006 3:04:43	41	36.7
4/14/2008 5:15:55	87	37.3	4/19/2007 2:42:34	64	36.9	4/23/2006 3:04:58	39	36.7
4/14/2008 5:16:00	88	37.3	4/19/2007 2:42:49	63	36.9	4/23/2006 3:05:13	37	36.7
4/14/2008 5:16:05	87	37.3	4/19/2007 2:43:04	61	36.9	4/23/2006 3:05:28	36	36.7
4/14/2008 5:16:10	86	37.3	4/19/2007 2:43:19	60	36.9	4/23/2006 3:05:43	35	36.7
4/14/2008 5:16:15	84	37.3	4/19/2007 2:43:34	60	36.9	4/23/2006 3:05:58	34	36.6
4/14/2008 5:16:20	82	37.3	4/19/2007 2:43:49	60	36.9	4/23/2006 3:06:13	32	36.7
4/14/2008 5:16:25	80	37.3	4/19/2007 2:44:04	60	36.9	4/23/2006 3:06:28	32	36.7
4/14/2008 5:16:30	77	37.3	4/19/2007 2:44:19	60	36.9	4/23/2006 3:06:43	31	36.7
4/14/2008 5:16:35	75	37.3	4/19/2007 2:44:34	58	36.9	4/23/2006 3:06:58	30	36.7
4/14/2008 5:16:40	73	37.3	4/19/2007 2:44:49	54	36.9	4/23/2006 3:07:13	29	36.6
4/14/2008 5:16:45	70	37.2	4/19/2007 2:45:04	51	36.9	4/23/2006 3:07:28	28	36.6
4/14/2008 5:16:50	69	37.3	4/19/2007 2:45:19	48	36.9	4/23/2006 3:07:43	27	36.6
4/14/2008 5:16:55	67	37.2	4/19/2007 2:45:34	46	36.9	4/23/2006 3:07:58	27	36.8
4/14/2008 5:17:00	66	37.2	4/19/2007 2:45:49	44	36.9	4/23/2006 3:08:13	26	36.9
4/14/2008 5:17:05	65	37.2	4/19/2007 2:46:04	42	36.9	4/23/2006 3:08:28	26	36.9
4/14/2008 5:17:10	63	37.2	4/19/2007 2:46:19	40	36.9	4/23/2006 3:08:43	27	36.9
4/14/2008 5:17:15	61	37.2	4/19/2007 2:46:34	38	36.9	4/23/2006 3:08:58	30	36.9
4/14/2008 5:17:20	61	37.2	4/19/2007 2:46:49	37	36.8	4/23/2006 3:09:13	32	36.9
4/14/2008 5:17:25	60	37.2	4/19/2007 2:47:04	36	36.8	4/23/2006 3:09:28	35	36.9
4/14/2008 5:17:30	59	37.2	4/19/2007 2:47:19	35	36.8	4/23/2006 3:09:43	38	36.9
4/14/2008 5:17:35	58	37.2	4/19/2007 2:47:34	33	36.8	4/23/2006 3:09:58	41	36.9
4/14/2008 5:17:40	57	37.2	4/19/2007 2:47:49	32	36.8	4/23/2006 3:10:13	43	36.9
4/14/2008 5:17:45	56	37.2	4/19/2007 2:48:04	31	36.8	4/23/2006 3:10:28	46	36.9
4/14/2008 5:17:50	56	37.2	4/19/2007 2:48:19	30	36.8	4/23/2006 3:10:43	48	36.9
4/14/2008 5:17:55	55	37.2	4/19/2007 2:48:34	29	36.8	4/23/2006 3:10:58	49	36.9
4/14/2008 5:18:00	54	37.2	4/19/2007 2:48:49	28	36.8	4/23/2006 3:11:13	50	36.9
4/14/2008 5:18:05	53	37.2	4/19/2007 2:49:04	28	36.8	4/23/2006 3:11:28	51	36.9
4/14/2008 5:18:10	53	37.2	4/19/2007 2:49:19	27	36.8	4/23/2006 3:11:43	51	36.8
4/14/2008 5:18:15	52	37.2	4/19/2007 2:49:34	27	36.8	4/23/2006 3:11:58	52	36.7
4/14/2008 5:18:20	52	37.2	4/19/2007 2:49:49	27	36.8	4/23/2006 3:12:13	52	36.7
4/14/2008 5:18:25	51	37.2	4/19/2007 2:50:04	27	36.8	4/23/2006 3:12:28	49	36.7
4/14/2008 5:18:30	51	37.2	4/19/2007 2:50:19	29	36.8	4/23/2006 3:12:43	46	36.7
4/14/2008 5:18:35	50	37.2	4/19/2007 2:50:34	31	36.8	4/23/2006 3:12:58	43	36.6
4/14/2008 5:18:40	50	37.2	4/19/2007 2:50:49	34	36.8	4/23/2006 3:13:13	41	36.7
4/14/2008 5:18:45	49	37.2	4/19/2007 2:51:04	36	36.8	4/23/2006 3:13:28	40	36.7
4/14/2008 5:18:50	49	37.2	4/19/2007 2:51:19	39	36.8	4/23/2006 3:13:43	38	36.7
4/14/2008 5:18:55	48	37.2	4/19/2007 2:51:34	43	36.8	4/23/2006 3:13:58	37	36.6
4/14/2008 5:19:00	48	37.2	4/19/2007 2:51:49	46	36.8	4/23/2006 3:14:13	35	36.7
4/14/2008 5:19:05	47	37.2	4/19/2007 2:52:04	50	36.8	4/23/2006 3:14:28	34	36.7
4/14/2008 5:19:10	47	37.2	4/19/2007 2:52:19	53	36.8	4/23/2006 3:14:43	33	36.7
4/14/2008 5:19:15	47	37.2	4/19/2007 2:52:34	56	36.8	4/23/2006 3:14:58	32	36.7
4/14/2008 5:19:20	46	37.2	4/19/2007 2:52:49	58	36.8	4/23/2006 3:15:13	32	36.6
4/14/2008 5:19:25	46	37.2	4/19/2007 2:53:04	61	36.8	4/23/2006 3:15:28	31	36.6
4/14/2008 5:19:30	45	37.2	4/19/2007 2:53:19	61	36.9	4/23/2006 3:15:43	30	36.6
4/14/2008 5:19:35	45	37.2	4/19/2007 2:53:34	62	36.9	4/23/2006 3:15:58	29	36.7
4/14/2008 5:19:40	44	37.2	4/19/2007 2:53:49	64	36.9	4/23/2006 3:16:13	28	36.6
4/14/2008 5:19:45	44	37.2	4/19/2007 2:54:04	63	36.9	4/23/2006 3:16:28	28	36.6
4/14/2008 5:19:50	44	37.2	4/19/2007 2:54:19	64	36.9	4/23/2006 3:16:43	27	36.6
4/14/2008 5:19:55	44	37.2	4/19/2007 2:54:34	64	36.9	4/23/2006 3:16:58	26	36.6
4/14/2008 5:20:00	43	37.2	4/19/2007 2:54:49	64	36.9	4/23/2006 3:17:13	25	36.6
4/14/2008 5:20:05	43	37.2	4/19/2007 2:55:04	63	36.9	4/23/2006 3:17:28	25	36.6

4/14/2008 5:20:10	43	37.2
4/14/2008 5:20:15	42	37.2
4/14/2008 5:20:20	42	37.2
4/14/2008 5:20:25	41	37.2
4/14/2008 5:20:30	41	37.2
4/14/2008 5:20:35	41	37.2
4/14/2008 5:20:40	40	37.2
4/14/2008 5:20:45	40	37.2
4/14/2008 5:20:50	40	37.2
4/14/2008 5:20:55	40	37.2
4/14/2008 5:21:00	39	37.2
4/14/2008 5:21:05	39	37.2
4/14/2008 5:21:10	39	37.2
4/14/2008 5:21:15	38	37.2
4/14/2008 5:21:20	38	37.2
4/14/2008 5:21:25	38	37.2
4/14/2008 5:21:30	38	37.2
4/14/2008 5:21:35	40	37.2
4/14/2008 5:21:40	42	37.2
4/14/2008 5:21:45	44	37.2
4/14/2008 5:21:50	46	37.2
4/14/2008 5:21:55	48	37.2
4/14/2008 5:22:00	49	37.2
4/14/2008 5:22:05	50	37.2
4/14/2008 5:22:10	52	37.2
4/14/2008 5:22:15	53	37.2
4/14/2008 5:22:20	55	37.2
4/14/2008 5:22:25	56	37.2
4/14/2008 5:22:30	58	37.2
4/14/2008 5:22:35	60	37.2
4/14/2008 5:22:40	61	37.2
4/14/2008 5:22:45	63	37.2
4/14/2008 5:22:50	65	37.2
4/14/2008 5:22:55	66	37.2
4/14/2008 5:23:00	67	37.2
4/14/2008 5:23:05	69	37.2
4/14/2008 5:23:10	71	37.2
4/14/2008 5:23:15	72	37.3
4/14/2008 5:23:20	74	37.2
4/14/2008 5:23:25	75	37.2
4/14/2008 5:23:30	76	37.3
4/14/2008 5:23:35	77	37.2
4/14/2008 5:23:40	77	37.2
4/14/2008 5:23:45	79	37.2
4/14/2008 5:23:50	80	37.2
4/14/2008 5:23:55	81	37.2
4/14/2008 5:24:00	81	37.3
4/14/2008 5:24:05	82	37.2
4/14/2008 5:24:10	84	37.2
4/14/2008 5:24:15	84	37.2
4/14/2008 5:24:20	85	37.3
4/14/2008 5:24:25	86	37.3
4/14/2008 5:24:30	86	37.3
4/14/2008 5:24:35	87	37.2
4/14/2008 5:24:40	86	37.2
4/14/2008 5:24:45	84	37.2
4/14/2008 5:24:50	84	37.2
4/14/2008 5:24:55	84	37.2
4/14/2008 5:25:00	85	37.3
4/14/2008 5:25:05	85	37.2
4/14/2008 5:25:10	86	37.2
4/14/2008 5:25:15	86	37.3
4/14/2008 5:25:20	87	37.3
4/14/2008 5:25:25	87	37.3
4/14/2008 5:25:30	86	37.3
4/14/2008 5:25:35	84	37.2
4/14/2008 5:25:40	82	37.2
4/14/2008 5:25:45	79	37.2
4/14/2008 5:25:50	76	37.2
4/14/2008 5:25:55	74	37.2
4/14/2008 5:26:00	72	37.2
4/14/2008 5:26:05	70	37.2
4/14/2008 5:26:10	68	37.2
4/14/2008 5:26:15	67	37.2
4/14/2008 5:26:20	65	37.2
4/14/2008 5:26:25	64	37.2
4/14/2008 5:26:30	63	37.2
4/14/2008 5:26:35	62	37.2
4/14/2008 5:26:40	60	37.2
4/14/2008 5:26:45	60	37.2
4/14/2008 5:26:50	58	37.2
4/14/2008 5:26:55	57	37.2
4/14/2008 5:27:00	57	37.2
4/14/2008 5:27:05	56	37.2
4/14/2008 5:27:10	55	37.2
4/14/2008 5:27:15	54	37.2
4/14/2008 5:27:20	54	37.2

4/19/2007 2:55:19	63	36.9
4/19/2007 2:55:34	64	36.9
4/19/2007 2:55:49	64	36.9
4/19/2007 2:56:04	64	36.9
4/19/2007 2:56:19	63	36.9
4/19/2007 2:56:34	63	36.9
4/19/2007 2:56:49	62	36.9
4/19/2007 2:57:04	61	36.9

Hepatic sinus apnea times

0:25:51	start
0:34:56	end
0:40:53	start
0:45:00	end
0:50:59	start
0:59:06	end
1:06:26	start
1:10:33	end
1:14:11	start
1:22:09	end
1:28:40	start
1:33:29	end
1:39:18	start
1:46:19	end
1:56:00	start
2:01:55	end
2:05:48	start
2:12:42	end
2:18:46	start
2:24:59	end
2:29:00	start
2:35:45	end
2:43:54	start
2:49:33	end

4/23/2006 3:17:43	24	36.6
4/23/2006 3:17:58	24	36.6
4/23/2006 3:18:13	23	36.6
4/23/2006 3:18:28	22	36.9
4/23/2006 3:18:43	22	36.9
4/23/2006 3:18:58	22	36.9
4/23/2006 3:19:13	24	36.9
4/23/2006 3:19:28	27	36.9
4/23/2006 3:19:43	29	36.9
4/23/2006 3:19:58	33	36.9
4/23/2006 3:20:13	36	36.9
4/23/2006 3:20:28	39	36.9
4/23/2006 3:20:43	42	36.9
4/23/2006 3:20:58	44	36.9
4/23/2006 3:21:13	45	36.9
4/23/2006 3:21:28	47	36.9
4/23/2006 3:21:43	48	36.9
4/23/2006 3:21:58	49	36.8
4/23/2006 3:22:13	48	37
4/23/2006 3:22:28	46	37
4/23/2006 3:22:43	46	36.9
4/23/2006 3:22:58	46	36.9
4/23/2006 3:23:13	48	36.9
4/23/2006 3:23:28	48	36.9
4/23/2006 3:23:43	49	36.8
4/23/2006 3:23:58	51	36.8
4/23/2006 3:24:13	50	36.7
4/23/2006 3:24:28	47	36.7
4/23/2006 3:24:43	45	36.7
4/23/2006 3:24:58	43	36.7
4/23/2006 3:25:13	41	36.7
4/23/2006 3:25:28	40	36.6
4/23/2006 3:25:43	38	36.6
4/23/2006 3:25:58	37	36.6
4/23/2006 3:26:13	35	36.6
4/23/2006 3:26:28	34	36.6
4/23/2006 3:26:43	33	36.6
4/23/2006 3:26:58	32	36.6
4/23/2006 3:27:13	31	36.6
4/23/2006 3:27:28	31	36.6
4/23/2006 3:27:43	30	36.6
4/23/2006 3:27:58	29	36.6
4/23/2006 3:28:13	28	36.6
4/23/2006 3:28:28	28	36.6
4/23/2006 3:28:43	27	36.6
4/23/2006 3:28:58	26	36.5
4/23/2006 3:29:13	25	36.6
4/23/2006 3:29:28	25	36.7
4/23/2006 3:29:43	24	36.9
4/23/2006 3:29:58	24	36.8
4/23/2006 3:30:13	24	36.9
4/23/2006 3:30:28	26	36.9
4/23/2006 3:30:43	29	36.9
4/23/2006 3:30:58	32	36.9
4/23/2006 3:31:13	35	36.9
4/23/2006 3:31:28	38	36.9
4/23/2006 3:31:43	41	36.9
4/23/2006 3:31:58	45	36.9
4/23/2006 3:32:13	47	36.9
4/23/2006 3:32:28	49	36.9
4/23/2006 3:32:43	51	36.9
4/23/2006 3:32:58	52	36.9
4/23/2006 3:33:13	53	36.9

Extradural vein apnea times

0:48:29	start
0:49:53	end
1:00:38	start
1:06:17	end
1:13:36	start
1:19:17	end
1:22:06	start
1:28:24	end
1:33:41	start
1:38:31	end
1:41:34	start
1:47:26	end
1:54:57	start
2:00:01	end
2:05:18	start
2:11:10	end
2:18:16	start
2:24:56	end

4/14/2008 5:27:25	53	37.2	2:30:25	start
4/14/2008 5:27:30	52	37.2	2:36:54	end
4/14/2008 5:27:35	52	37.2	2:45:25	start
4/14/2008 5:27:40	51	37.2	2:51:05	end
4/14/2008 5:27:45	51	37.2	2:54:32	start
4/14/2008 5:27:50	50	37.2	3:01:01	end
4/14/2008 5:27:55	50	37.2	3:03:52	start
4/14/2008 5:28:00	49	37.2	3:08:19	end
4/14/2008 5:28:05	49	37.2	3:11:57	start
4/14/2008 5:28:10	48	37.2	3:18:51	end
4/14/2008 5:28:15	48	37.2	3:23:55	start
4/14/2008 5:28:20	47	37.2	3:29:59	end
4/14/2008 5:28:25	47	37.2		
4/14/2008 5:28:30	46	37.2		
4/14/2008 5:28:35	46	37.2		
4/14/2008 5:28:40	46	37.2		
4/14/2008 5:28:45	45	37.2		
4/14/2008 5:28:50	45	37.2		
4/14/2008 5:28:55	45	37.2		
4/14/2008 5:29:00	44	37.2		
4/14/2008 5:29:05	44	37.2		
4/14/2008 5:29:10	43	37.2		
4/14/2008 5:29:15	43	37.2		
4/14/2008 5:29:20	43	37.2		
4/14/2008 5:29:25	42	37.2		
4/14/2008 5:29:30	42	37.2		
4/14/2008 5:29:35	42	37.2		
4/14/2008 5:29:40	41	37.2		
4/14/2008 5:29:45	41	37.2		
4/14/2008 5:29:50	41	37.2		
4/14/2008 5:29:55	40	37.2		
4/14/2008 5:30:00	40	37.2		
4/14/2008 5:30:05	40	37.2		
4/14/2008 5:30:10	39	37.2		
4/14/2008 5:30:15	39	37.2		
4/14/2008 5:30:20	39	37.2		
4/14/2008 5:30:25	39	37.2		
4/14/2008 5:30:30	38	37.2		
4/14/2008 5:30:35	38	37.1		
4/14/2008 5:30:40	38	37.1		
4/14/2008 5:30:45	38	37.2		
4/14/2008 5:30:50	40	37.2		
4/14/2008 5:30:55	42	37.2		
4/14/2008 5:31:00	44	37.2		
4/14/2008 5:31:05	47	37.2		
4/14/2008 5:31:10	49	37.2		
4/14/2008 5:31:15	51	37.2		
4/14/2008 5:31:20	53	37.2		
4/14/2008 5:31:25	55	37.2		
4/14/2008 5:31:30	56	37.2		
4/14/2008 5:31:35	58	37.2		
4/14/2008 5:31:40	60	37.2		
4/14/2008 5:31:45	61	37.2		
4/14/2008 5:31:50	63	37.2		
4/14/2008 5:31:55	64	37.2		
4/14/2008 5:32:00	66	37.2		
4/14/2008 5:32:05	68	37.2		
4/14/2008 5:32:10	70	37.2		
4/14/2008 5:32:15	71	37.2		
4/14/2008 5:32:20	73	37.2		
4/14/2008 5:32:25	74	37.2		
4/14/2008 5:32:30	76	37.2		
4/14/2008 5:32:35	78	37.2		
4/14/2008 5:32:40	78	37.2		
4/14/2008 5:32:45	79	37.2		
4/14/2008 5:32:50	81	37.2		
4/14/2008 5:32:55	82	37.2		
4/14/2008 5:33:00	83	37.2		
4/14/2008 5:33:05	85	37.2		
4/14/2008 5:33:10	86	37.2		
4/14/2008 5:33:15	86	37.2		
4/14/2008 5:33:20	87	37.2		
4/14/2008 5:33:25	87	37.2		
4/14/2008 5:33:30	88	37.2		
4/14/2008 5:33:35	88	37.2		
4/14/2008 5:33:40	89	37.2		
4/14/2008 5:33:45	89	37.2		
4/14/2008 5:33:50	89	37.2		
4/14/2008 5:33:55	88	37.3		
4/14/2008 5:34:00	88	37.2		
4/14/2008 5:34:05	88	37.2		
4/14/2008 5:34:10	88	37.2		
4/14/2008 5:34:15	88	37.2		
4/14/2008 5:34:20	88	37.2		
4/14/2008 5:34:25	89	37.2		
4/14/2008 5:34:30	90	37.2		
4/14/2008 5:34:35	90	37.2		

4/14/2008 5:34:40	91	37.2
4/14/2008 5:34:45	91	37.2
4/14/2008 5:34:50	92	37.2
4/14/2008 5:34:55	92	37.2
4/14/2008 5:35:00	92	37.2
4/14/2008 5:35:05	93	37.2
4/14/2008 5:35:10	95	37.2
4/14/2008 5:35:15	96	37.3
4/14/2008 5:35:20	96	37.3
4/14/2008 5:35:25	97	37.2
4/14/2008 5:35:30	96	37.2
4/14/2008 5:35:35	96	37.2
4/14/2008 5:35:40	95	37.2
4/14/2008 5:35:45	95	37.2
4/14/2008 5:35:50	95	37.2
4/14/2008 5:35:55	94	37.2
4/14/2008 5:36:00	93	37.2
4/14/2008 5:36:05	93	37.2
4/14/2008 5:36:10	93	37.2
4/14/2008 5:36:15	94	37.3
4/14/2008 5:36:20	95	37.3
4/14/2008 5:36:25	95	37.2
4/14/2008 5:36:30	94	37.3
4/14/2008 5:36:35	94	37.3
4/14/2008 5:36:40	94	37.2
4/14/2008 5:36:45	94	37.3
4/14/2008 5:36:50	93	37.3
4/14/2008 5:36:55	93	37.2
4/14/2008 5:37:00	92	37.2
4/14/2008 5:37:05	92	37.2
4/14/2008 5:37:10	92	37.2
4/14/2008 5:37:15	92	37.2
4/14/2008 5:37:20	92	37.2
4/14/2008 5:37:25	92	37.2
4/14/2008 5:37:30	93	37.2
4/14/2008 5:37:35	94	37.3
4/14/2008 5:37:40	95	37.3
4/14/2008 5:37:45	95	37.3
4/14/2008 5:37:50	96	37.3
4/14/2008 5:37:55	96	37.3
4/14/2008 5:38:00	97	37.3
4/14/2008 5:38:05	97	37.3
4/14/2008 5:38:10	97	37.3
4/14/2008 5:38:15	96	37.3
4/14/2008 5:38:20	96	37.3
4/14/2008 5:38:25	95	37.3
4/14/2008 5:38:30	94	37.3
4/14/2008 5:38:35	93	37.3
4/14/2008 5:38:40	92	37.3
4/14/2008 5:38:45	92	37.3
4/14/2008 5:38:50	91	37.3
4/14/2008 5:38:55	90	37.3
4/14/2008 5:39:00	89	37.3
4/14/2008 5:39:05	105	37.3
4/14/2008 5:39:10	99	37.3
4/14/2008 5:39:15	96	37.3
4/14/2008 5:39:20	91	37.3
4/14/2008 5:39:25	90	37.3
4/14/2008 5:39:30	91	37.3
4/14/2008 5:39:35	91	37.3
4/14/2008 5:39:40	89	37.3
4/14/2008 5:39:45	88	37.3
4/14/2008 5:39:50	90	37.4
4/14/2008 5:39:55	90	37.4
4/14/2008 5:40:00	90	37.4

Arterial apnea times

4:42:43	start
4:48:07	end
4:55:55	start
5:02:05	end
5:06:32	start
5:10:37	end
5:16:08	start
5:21:13	end
5:25:24	start
5:30:30	end
